# Correction: Evaluation of the shielding initiative in Wales (EVITE Immunity): protocol for a quasiexperimental study

**DOI:** 10.1136/bmjopen-2021-059813corr1

**Published:** 2022-12-23

Evans BA, Akbari A, Bailey R, *et al*. Evaluation of the shielding initiative in Wales (EVITE Immunity): protocol for a quasiexperimental study. *BMJ Open* 2022;0:e059813. doi:10.1136/bmjopen-2021-059813

This article has been corrected since it was published online. A new author “Victoria Williams” has been added to the author byline.

