# Supplementary material for: Evaluation of the shielding initiative in Wales (EVITE Immunity): protocol for a quasiexperimental study
Source: BMJ Open. 2022 Sep 8;12(9):e059813. doi: 10.1136/bmjopen-2021-059813 (PMC9461087; doi:10.1136/bmjopen-2021-059813)
Supplement: Supplementary data [file bmjopen-2021-059813supp001.pdf]

Appendix 1

EVITE Immunity

Prifysgol Abertawe Swansea University | Ysgol Feddygaeth Medical School

GIG CYMRU NHS WALES | Iechyd a Gofal Digidol Cymru Digital Health and Care Wales

Address line 1
Address line 2
Address line 3
Address line 4
Address line 5

Dear [example – Mr David Jones –]

Please cut or tear to remove the **top** section of **this page** before returning

(your study ID is 12345)

We would like to invite you to help us with our research study looking at the effects of public health policy in Wales during the COVID-19 pandemic.

We are asking you to help by completing the questionnaire and sending it back to us using the enclosed prepaid envelope. We would like to know about how you have been feeling over the last 12 months. The questionnaire should take around 20 minutes to complete. If you are happy to take part, please complete the questionnaire and return to the researchers using the supplied prepaid envelope. You can use the QR code to complete it online or by going to the website address below. We are not able to provide the questionnaire in Welsh as some parts of it have only been tested and validated for research in English.

Your input in this study is really valuable and we appreciate your time in completing the questionnaire.

Kind regards,

Professor Helen Snooks, Chief Investigator, Dr Victoria Williams, Study Manager, and the EVITE Immunity Study Team

If you would prefer to complete the questionnaire online, please scan in the QR code or visit the following website:
https://swansea.onlinesurveys.ac.uk/evite-immunity-questionnaire-su

QR CODE TO ONLINE SURVEY

Please contact us for further information or assistance to complete the questionnaire
Phone: 01792 513279
Email: EVITEIMMUNITY2@Swansea.ac.uk

1

IRAS 295050

BMJ Publishing Group Limited (BMJ) disclaims all liability and responsibility arising from any reliance placed on this supplemental material which has been supplied by the author(s)

Annwyl Sir/madam

Hoffem eich gwahodd i'n helpu ni gyda’n hastudiaeth ymchwil sy'n ystyried effeithiau polisi iechyd cyhoeddus yng Nghymru yn ystod pandemig Covid-19.

Gofynnwn i chi ein helpu ni trwy gwblhau'r holiadur a'i ddychwelyd atom ni gan ddefnyddio'r amlen wedi’i rhagdalu amgaeedig. Hoffem wybod sut rydych chi wedi bod yn teimlo dros y 12 mis diwethaf. Dylai gymryd tuag 20 munud i gwblhau'r holiadur. Os ydych chi'n hapus i gymryd rhan, llenwch yr holiadur a'i ddychwelyd at yr ymchwilwyr gan ddefnyddio'r amlen wedi’i rhagdalu amgaeedig. Gallwch chi ddefnyddio'r côd QR i'w lenwi ar-lein neu drwy fynd i gyfeiriad y wefan isod. Ni allwn ddarparu'r holiadur yn Gymraeg am fod rhai rhannau wedi'u profi a'u dilysu ar gyfer ymchwil yn Saesneg yn unig.

Mae eich mewnbwn i'r astudiaeth hon yn werthfawr iawn ac rydym yn gwerthfawrogi'ch amser yn llenwi'r holiadur.

Cofion cynnes,

Yr Athro Helen Snooks, Prif Ymchwilydd, Dr Victoria Williams, Rheolwr yr Astudiaeth, a Thîm Astudiaeth Imiwnedd EVITE

Os byddai’n well gennych chi lenwi'r holiadur ar-lein, sganiwch y côd QR neu ewch i'r wefan ganlynol: https://swansea.onlinesurveys.ac.uk/evite-immunity-questionnaire-su

CÔD QR AR GYFER YR AROLWG AR-LEIN

Cysylltwch â ni am gymorth neu wybodaeth pellach i'w gwbwlhau yr ymholiadur os gwelwch yn dda.

Ffôn: 01792 513279

E-bost: EVITEIMMUNITY2@Swansea.ac.uk

Evans BA, *et al. BMJ Open* 2022; 12:e059813. doi: 10.1136/bmjopen-2021-059813

Study ID Number: | | | | | |

[Please insert your Study ID as shown on the first page of the cover letter]

Section 1

1. Did you at any time receive a shielding letter or text message from the NHS or Chief Medical Officer saying that you have been identified as someone at risk of severe illness (COVID-19) if you catch Coronavirus, because you have an underlying disease or health condition? Please select a box that applies.

Yes	
No	
Don't know	
Don't want to answer	

2. **After the first lockdown was announced in March 2020,** on average can you recall how many days you met up with any of your family or friends who do not live with you?

Everyday	
4-6 days each week	
2-3 days each week	
1 day each week	
Less than 1 day per week	
Don't know	
Don't want to answer	

3. **Over the last 2 weeks,** on average can you recall how many days you met up with any of your family or friends who do not live with you?

Everyday	
4-6 days each week	
2-3 days each week	
1 day each week	
Less than 1 day per week	
Don't know	
Don't want to answer	

Evans BA, *et al. BMJ Open* 2022; 12:e059813. doi: 10.1136/bmjopen-2021-059813

4. The following questions are about what you did at the time of the initial announcement of lockdown in March and during the last 2 weeks. Please select one response for each question and each time point (i.e. March and the last 2 weeks)

On a scale of 1 to 5, where 1 is Never and 5 is Always, please let us know how often you did the below.

	**During the initial announcement of lockdown in March 2020**	**During the last 2 weeks**
Strictly avoided contact with someone who displayed symptoms of coronavirus	1. Never<br>2. Rarely<br>3. Sometimes<br>4. Very often<br>5. Always	1. Never<br>2. Rarely<br>3. Sometimes<br>4. Very often<br>5. Always
Stayed at home	1. Never<br>2. Rarely<br>3. Sometimes<br>4. Very often<br>5. Always	1. Never<br>2. Rarely<br>3. Sometimes<br>4. Very often<br>5. Always
Felt scared to go outside	1. Never<br>2. Rarely<br>3. Sometimes<br>4. Very often<br>5. Always	1. Never<br>2. Rarely<br>3. Sometimes<br>4. Very often<br>5. Always
Attended any gathering (Including gatherings of friends and families in private spaces e.g. family homes, weddings and religious services)	1. Never<br>2. Rarely<br>3. Sometimes<br>4. Very often<br>5. Always	1. Never<br>2. Rarely<br>3. Sometimes<br>4. Very often<br>5. Always
Went out for shopping, leisure or travel	1. Never<br>2. Rarely<br>3. Sometimes<br>4. Very often<br>5. Always	1. Never<br>2. Rarely<br>3. Sometimes<br>4. Very often<br>5. Always
Kept in touch using remote technology e.g. phone, internet and social media	1. Never<br>2. Rarely<br>3. Sometimes<br>4. Very often<br>5. Always	1. Never<br>2. Rarely<br>3. Sometimes<br>4. Very often<br>5. Always
Used telephone or online services to contact your GP or other essential services	1. Never<br>2. Rarely<br>3. Sometimes<br>4. Very often<br>5. Always	1. Never<br>2. Rarely<br>3. Sometimes<br>4. Very often<br>5. Always
Regularly washed your hands with soap and water for 20 seconds	1. Never<br>2. Rarely<br>3. Sometimes<br>4. Very often<br>5. Always	1. Never<br>2. Rarely<br>3. Sometimes<br>4. Very often<br>5. Always

Evans BA, *et al. BMJ Open* 2022; 12:e059813. doi: 10.1136/bmjopen-2021-059813

5. The following questions are about what you did after the initial announcement of lockdown in March and during the last 2 weeks. Please select one response for each question and each time point (i.e. March and the last 2 weeks)

On a scale of 1 to 5 , where 1 is Never and 5 is Always, please let us know how often you did the below within your household (including visitors and carers)

	**During the initial announcement of lockdown in March 2020**	**During the last 2 weeks**
Minimised the time you spent with others from your household in shared spaces (kitchen, bathroom and sitting areas)	1. Never<br>2. Rarely<br>3. Sometimes<br>4. Very often<br>5. Always	1. Never<br>2. Rarely<br>3. Sometimes<br>4. Very often<br>5. Always
Kept shared spaces well ventilated	1. Never<br>2. Rarely<br>3. Sometimes<br>4. Very often<br>5. Always	1. Never<br>2. Rarely<br>3. Sometimes<br>4. Very often<br>5. Always
Used separate towels from the rest of your household	1. Never<br>2. Rarely<br>3. Sometimes<br>4. Very often<br>5. Always	1. Never<br>2. Rarely<br>3. Sometimes<br>4. Very often<br>5. Always
Used a separate bathroom from the rest of your household or cleaned the bathroom after every use	1. Never<br>2. Rarely<br>3. Sometimes<br>4. Very often<br>5. Always	1. Never<br>2. Rarely<br>3. Sometimes<br>4. Very often<br>5. Always
Avoided using the kitchen when others are present and ensured kitchenware is cleaned thoroughly	1. Never<br>2. Rarely<br>3. Sometimes<br>4. Very often<br>5. Always	1. Never<br>2. Rarely<br>3. Sometimes<br>4. Very often<br>5. Always

The following questions are to help us understand any safety concerns you have had over the last 12 months.

6. **While trying to access or receive NHS care during the coronavirus pandemic have you experienced something that you thought was a 'safety concern'?**

A safety concern can be any event or situation where you think something went wrong or not as good as expected whilst receiving or trying to receive health care. The event or situation might have led to harm or you feel could result in harm in the future if the concern is not addressed. By harm, we mean the event or situation is impacting you or another person physically (like a worsening of symptoms or your condition, or any disabling complications), emotionally (upset or loss of trust) or psychologically (any worsening of your mental health).

Yes	
No	
Don't know	
Don't want to answer	

IF YES GO TO **QUESTION 7**, IF NO GO TO **SECTION 2.**

7. **What did the safety concerns relate to and on a scale from 1 (not serious at all) to 10 (extremely serious), how serious do you think your safety concern was? Please rate any concern that you experienced by selecting the appropriate number on the scale.**

Diagnosis of your problem	1 2 3 4 5 6 7 8 9 10
Access to the NHS service you needed	1 2 3 4 5 6 7 8 9 10
Tests or procedures that were performed (e.g. blood tests, scans)	1 2 3 4 5 6 7 8 9 10
Medication, vaccines or treatment	1 2 3 4 5 6 7 8 9 10
Delay or cancellation of treatment for pre-existing condition	1 2 3 4 5 6 7 8 9 10
Communication between you and the healthcare professional(s)	1 2 3 4 5 6 7 8 9 10
Communication and co-ordination between different healthcare professionals	1 2 3 4 5 6 7 8 9 10
Concerns specific to the coronavirus outbreak (e.g. personal protective equipment)	1 2 3 4 5 6 7 8 9 10
Information provided	1 2 3 4 5 6 7 8 9 10
Vaccination	1 2 3 4 5 6 7 8 9 10
Other	1 2 3 4 5 6 7 8 9 10
If other, please specify ____________________________.	1 2 3 4 5 6 7 8 9 10

Evans BA, *et al. BMJ Open* 2022; 12:e059813. doi: 10.1136/bmjopen-2021-059813

8. **In which health care setting(s) did the safety concern(s) take place? Please tick all that apply**.

GP services (e.g. GP, nurse appointment, health visitor)	
A&E	
Routine outpatient services (e.g. appointment with hospital doctor/nurse specialist, consultant, physiotherapy, speech therapy, dialysis, mental health services)	
Inpatient services (e.g. routine surgery, admission to hospital)	
Midwifery and maternity services	
District nurse	
Optician	
Pharmacist	
Dentist	
NHS 111 service	
COVID-19 Vaccination services	
Other	
If other, please specify	

9. **Is there anything else that you would like to tell us about your safety concern(s)?**

Yes	
No	
If yes, please specify	_________

If you would like advice on reporting a patient safety concern, please visit:
Wales: https://www.avma.org.uk/wp-content/uploads/Complaints-Wales.pdf

Section 2

Over the last two weeks, how often have you been bothered by any of the following problems?

	Not at all	Several Days	More than half the days	Nearly everyday
Little interest of pleasure in doing things				
Feeling down, depressed, or hopeless				
Trouble falling or staying asleep, or sleeping too much				
Feeling tired or having little energy				
Poor appetite or overeating				
Feeling bad about yourself-or that you are a failure or have let yourself or your family down				
Trouble concentrating on things, such as reading the newspaper or watching television				
Moving or speaking so slowly that other people could have noticed? Or the opposite- being so fidgety or restless that you have been moving around a lot more than usual				
Thoughts that you would be better off dead, or of hurting yourself in some way				

Section 3

Over the last 2 weeks, how often have you been bothered by any of the following problems?

	Not at all	Several Days	More than half the days	Nearly everyday
Feeling nervous, anxious or on edge				
Not being able to stop or control worrying				
Worrying too much about different things				
Trouble relaxing				
Being so restless that it is hard to sit still				
Becoming easily annoyed or irritable				
Feeling afraid as if something awful might happen				

Section 4

This section of the questionnaire asks for your views about your health. This information will help keep track of how you feel and how well you are able to do your usual activities. We will ask you how you have felt at different time points – two weeks ago and four weeks ago **Answer each question by choosing just one answer.** If you are unsure how to answer a question, please give the best answer you can.

1. **In general, would you say your health is:**

Excellent ☐

Very good ☐

Good ☐

Fair ☐

Poor ☐

The following questions are about activities you might do during a typical day. Does your health now limit you in these activities? If so, how much?

	YES, limited a lot	YES, limited a little	NO, not limited at all
2. **Moderate activities** such as digging in the garden, spring cleaning or other heavy housework, gentle swimming or cycling	☐	☐	☐
3. Climbing **several** flights of stairs	☐	☐	☐

During the past 4 weeks, have you had any of the following problems with your work or other regular daily activities as a result of your physical health?

	YES	NO
4. **Accomplished** less than you would like	☐	☐
5. Were **limited** in the kind of work or other activities	☐	☐

Evans BA, *et al. BMJ Open* 2022; 12:e059813. doi: 10.1136/bmjopen-2021-059813

During the past 4 weeks have you had any of the following problems with your work or other regular daily activities as a result of any emotional problems (such as feeling depressed or anxious)?

	**YES**	**NO**
6. **Accomplished** less than you would like	☐	☐
7. Did work or activities **less carefully than usual**	☐	☐

8. During the past 4 weeks, how much did pain interfere with your normal work (including work outside the home and housework)?

Not at all ☐

A little bit ☐

Moderately ☐

Quite a bit ☐

Extremely ☐

These questions are about how you have been feeling during the past 4 weeks. For each question, please give the one answer that comes closest to the way you have been feeling.

How much of the time during the past 4 weeks…

	**All of the time**	**Most of the time**	**A good bit of the time**	**Some of the time**	**A little of the time**	**None of the time**
9. Have you felt calm and peaceful?	☐	☐	☐	☐	☐	☐
10. Did you have a lot of energy?	☐	☐	☐	☐	☐	☐
11. Have you felt down-hearted and blue?	☐	☐	☐	☐	☐	☐

Evans BA, *et al. BMJ Open* 2022; 12:e059813. doi: 10.1136/bmjopen-2021-059813

12. **During the past 4 weeks, how much of the time has your physical health or emotional problems interfered with your social activities (like visiting friends, relatives, etc)?**

All of the time ☐

Most of the time ☐

Some of the time ☐

A little of the time ☐

None of the time ☐

Section 5

Please indicate how often each of the statements below is descriptive of you:

	**I often feel this way**	**I sometimes feel this way**	**I rarely feel this way**	**I never feel this way**
**I lack companionship**	☐	☐	☐	☐
**I feel part of a group of friends**	☐	☐	☐	☐
**I feel left out**	☐	☐	☐	☐
**I feel isolated**	☐	☐	☐	☐
**I am unhappy being so withdrawn**	☐	☐	☐	☐
**People are around me but not with me**	☐	☐	☐	☐

Section 6

The following questions ask you about treatment for any infections you contracted during the past two years, before and during the pandemic.

1. Thinking back to March 2019 - March 2020, the year before the pandemic, how many times do you estimate you took a course of antibiotics?

	Please tick which applies
0	
1 – 3 times	
4 – 6 times	
7 – 10 times	
More than 10 times	
Part of daily/weekly medication regime	
Other (please give details)	

2. Thinking back to March 2020 – March 21, the year since the pandemic started, how many times do you estimate you took a course of antibiotics?

	Please tick which applies
0	
1 – 3 times	
4 – 6 times	
7 – 10 times	
More than 10 times	
Part of daily/weekly medication regime	
Other (please give details)	

Section 7

Please let us know if you have anything else you would like to say about your experience over the last 12 months

Have you had at least one COVID-19 vaccination?

Yes ☐

No ☐

Thank you for your time in completing this questionnaire. Your input is really valuable. Please could you provide us with some information on who completed the questionnaire:

1. I completed the questionnaire without assistance ☐
2. A carer helped me to complete the questionnaire ☐

Further Research

Your answers will be treated in the strictest confidence and will only be used for research purposes. Your responses may be used with other anonymised information about you. *If you do not want your data to be used in this way please tick this box:*

☐

Please let us know if you are happy for us to contact you regarding other aspects of this research. Please tick the below boxes if you give your consent for us to contact you regarding:

1. Taking blood samples ☐
2. Telephone interviews ☐

If you consented to either or both of the above please provide appropriate contact details:

Telephone number: ________________________

Email address: ______________________________________
