# Supplementary material for: Evaluation of the shielding initiative in Wales (EVITE Immunity): protocol for a quasiexperimental study
Source: BMJ Open. 2022 Sep 8;12(9):e059813. doi: 10.1136/bmjopen-2021-059813 (PMC9461087; doi:10.1136/bmjopen-2021-059813)
Supplement: Supplementary data [file bmjopen-2021-059813supp002.pdf]

Appendix 3

Effects of shielding for Vulnerable people during COVID-19 pandemic on health outcomes, costs and Immunity including those with cancer: quasi-experimenTal Evaluation (EVITE Immunity)

Healthcare Professionals
Interview questions - v1.0 10.05.21

1. What is your clinical role?
    - GP/hospital doctor – speciality/nurse/other

2. How were you first informed about the shielding programme for patients extremely vulnerable to Covid-19?
    - Letter/other contact
    - Was it clear?
    - Any change in guidance over time? Have you been kept informed of changes?

3. What has been your role in putting the shielding programme in place?
    - Contribution to designing it
    - Selecting patients at risk- criteria for including or excluding people from the list and whether this changed
    - Providing feedback
    - Putting patients in touch with support (eg food parcels)

4. What do you think of the shielding programme?
    - Has it been implemented as planned?
    - Positive aspects – for patients, for health service
    - Negative aspects – for patients, for health service
    - Have things changed over time?

      How did the way you delivered healthcare change for patients who were shielding?

5. We have already talked to patients who were part of the shielding programme. Here are two of their stories. *Share written vignettes, with names/details changed.*
    - Do these resonate with you? Is there anything in them which surprises you?
    - Have you discussed the shielding programme with any of your patients? What did they tell you?

IRAS 295050
EVITE Immunity interviews with HCPs v1.0 10.05.21

EVITE Immunity

Effects of shielding for Vulnerable people during COVID-19 pandemic on health and Immunity: a quasi-experimenTal Evaluation

Swansea University
Prifysgol Abertawe
Medical School
Ysgol Feddygaeth

6. Are you aware of any health related risks that your patients have experienced associated with the shielding programme?

7. Do you have any suggestions for any way it could be improved?

IRAS 295050
EVITE Immunity interviews with HCPs v1.0 10.05.21
