# Supplementary material for: Evaluation of the shielding initiative in Wales (EVITE Immunity): protocol for a quasiexperimental study
Source: BMJ Open. 2022 Sep 8;12(9):e059813. doi: 10.1136/bmjopen-2021-059813 (PMC9461087; doi:10.1136/bmjopen-2021-059813)
Supplement: Supplementary data [file bmjopen-2021-059813supp003.pdf]

Appendix 2

EVITE Immunity

Effects of shielding for Vulnerable people during COVID-19 pandemic on health and Immunity: a quasi-experimenTal Evaluation

Swansea University
Prifysgol Abertawe
Medical School
Ysgol Feddygaeth

Effects of shielding for Vulnerable people during COVID-19 pandemic on health outcomes, costs and Immunity including those with cancer: quasi-experimenTal Evaluation (EVITE Immunity)

Shielded population-interview questions- v1.0 10.05.21

Establish before the interview whether the person responding is someone who has been shielding, a carer, or both are being interviewed at once.

1. Can you tell me about your household please?
   - Live alone?
   - Live with others? How many/who?
   - Anyone else in your household also shielding?
     i. Were these other people also sent a shielding letter
     ii. were they family members who decided to shield with you
   - Does anyone regularly come to your home to provide care or support? If so, paid carer or informal/family carer?

2. Can you recall receiving the letter about shielding in March or April 2020?
   - Was it clear to you?
   - How did the letter make you feel?

3. Since April 2020, have you received any more information about shielding?
   - Letter/other contact
   - Any change in guidance?

4. Once you got the shielding letter, how did your life change?
   - Staying in?
   - Avoiding visitors?
   - Avoiding interaction with other household members?
   - What stayed the same for you after you got the shielding letter
   - What aspects of your life were better because you were shielding?

5. It has been more than a year since the shielding programme was introduced. Over that time, have you made any changes in how you have lived, eg starting to go out of the house more.
   - When did you change what you were doing? (clarify how long they felt they were shielding)
   - Why did you change?
   - Have things changed more than once – eg going out more in the summer, staying home more in the winter, influence of changing rules and lockdowns?
   - Do you behave differently now that shielding has ended,compared to before the pandemic?

IRAS 295050
EVITE Immunity interviews with shielded population v1.0 10.05.21

6. How have you felt about being part of the shielding programme?
    - Positive feelings – eg Safe, protected, doing your bit
    - Negative feelings – eg frustrated, confused, afraid, lonely, sad
    - Neutral - took no notice of it
    - Have your feelings changed over time?
    - Do you consider yourself to have 'been shielding yourself' or 'being shielded by others/community'?

7. If you live with other people, what impact has the shielding programme had on them?
    - Have they changed the way they behave?
    - Explore whether others in household shielded/did not shield; why did they make this decision; what difference did it make for you?

8. Did you get any additional support to help you with shielding?
    - eg food parcels, help from your employer

9. Have you had contact with the health service over the last year (GP/Hospital/NHS 111/ambulance service etc)?
    - To discuss Covid 19 risks/shielding
    - For other health reasons
    - Hesitant about contacting NHS/avoided contact
    - Any cancelled or delayed treatments or tests
    - How has your normal healthcare changed over the past year?

10. Do you know anyone who has had Covid 19?

    - Self
    - Someone they have had direct contact with in the last year
    - Someone they have not had contact with in the last year
    - If yes, did that affect how they felt about shielding?
