# Supplementary material for: Evaluation of the shielding initiative in Wales (EVITE Immunity): protocol for a quasiexperimental study
Source: BMJ Open. 2022 Sep 8;12(9):e059813. doi: 10.1136/bmjopen-2021-059813 (PMC9461087; doi:10.1136/bmjopen-2021-059813)
Supplement: Author's manuscript [file bmjopen-2021-059813.draft_revisions.pdf]

BMJ Open

BMJ Open is committed to open peer review. As part of this commitment we make the peer review history of every article we publish publicly available.

When an article is published we post the peer reviewers' comments and the authors' responses online. We also post the versions of the paper that were used during peer review. These are the versions that the peer review comments apply to.

The versions of the paper that follow are the versions that were submitted during the peer review process. They are not the versions of record or the final published versions. They should not be cited or distributed as the published version of this manuscript.

BMJ Open is an open access journal and the full, final, typeset and author-corrected version of record of the manuscript is available on our site with no access controls, subscription charges or pay-per-view fees (http://bmjopen.bmj.com).

If you have any questions on BMJ Open's open peer review process please email info.bmjopen@bmj.com

BMJ Open

Evaluation of the Shielding Initiative in Wales (EVITE Immunity):Protocol for a quasi-experimental study

Journal:	*BMJ Open*
Manuscript ID	bmjopen-2021-059813
Article Type:	Protocol
Date Submitted by the Author:	13-Dec-2021
Complete List of Authors:	Evans, Bridie; Swansea University Medical School; Swansea University Medical School, PRIME Centre Wales<br>Akbari, Ashley; Swansea University Medical School<br>Bailey, Rowena; Swansea University Medical School<br>Bethell, Lesley; Swansea University Medical School, public contributor<br>Bufton, Samantha; Welsh Government<br>Carson-Stevens, Andrew; Cardiff University<br>Dixon, Lucy; Swansea University Medical School, public contributor<br>Edwards, Adrian; Cardiff University<br>John, Ann; Swansea University Medical School<br>Jolles, Stephen; University Hospital of Wales<br>Kingston, Mark; Swansea University Medical School; Swansea University Medical School, PRIME Centre Wales<br>Lyons, Jane; Swansea University Medical School<br>Lyons, Ronan; Swansea University Medical School<br>Porter, Alison; Swansea University Medical School; Swansea University Medical School, PRIME Centre Wales<br>Sewell, Bernadette; Swansea University, Swansea Centre for Health Economics<br>Watkins, Alan; Swansea University Medical School<br>Whiffen, Tony; Welsh Government<br>Snooks, Helen; Swansea University Medical School; Swansea University Medical School, PRIME Centre Wales
Keywords:	COVID-19, IMMUNOLOGY, PUBLIC HEALTH, Health policy < HEALTH SERVICES ADMINISTRATION & MANAGEMENT

SCHOLARONE™
Manuscripts

Evaluation of the Shielding Initiative in Wales (EVITE Immunity): Protocol for a quasi-experimental study

Authors:

Bridie Angela Evans [1,2*]

Ashley Akbari [1,3]

Rowena Bailey [1,3]

Lesley Bethell [4]

Samantha Bufton [5]

Andrew Carson-Stevens [6]

Lucy Dixon [4]

Adrian Edwards [6]

Ann John [1,3]

Stephen Jolles [7]

Mark Kingston[1, 2]

Jane Lyons [1,3]

Ronan A Lyons [1,3]

Alison Porter [1,2]

Bernadette Sewell [8]

Catherine A Thornton [1]

Alan Watkins[1]

Anthony Whiffen [5]

Helen Snooks [1,2]

Affiliations:

[1] Swansea University Medical School, Swansea, UK

[2] PRIME Centre Wales, Swansea University Medical School, Swansea, UK

[3] Population Data Science, Health Data Research UK, Swansea University, Swansea, UK

[4] Public contributor, c/o Swansea University Medical School, Swansea, UK

[5] Administrative Data Research Unit, Welsh Government, Cardiff, UK

For peer review only

For peer review only - http://bmjopen.bmj.com/site/about/guidelines.xhtml

[6] PRIME Centre Wales, Division of Population Medicine, School of Medicine, Cardiff University, Cardiff, UK

[7] Immunodeficiency Centre for Wales, University Hospital of Wales, Cardiff, UK

[8] Swansea Centre for Health Economics, Swansea University, Swansea, UK

***Corresponding author:**

Bridie Angela Evans

Institute of Life Science

Swansea University Medical School

Singleton Campus

Swansea SA2 8PP

Authors' email addresses:

b.a.evans@swansea.ac.uk

A.Akbari@Swansea.ac.uk

R.Bailey@Swansea.ac.uk

lesleybethell@gmail.com

Samantha.Bufton@gov.wales

andypcs@gmail.com

lucydixon40@gmail.com

EdwardsAG@cardiff.ac.uk

A.John@Swansea.ac.uk

JollesSR@cardiff.ac.uk

M.R.Kingston@Swansea.ac.uk

J.Lyons@Swansea.ac.uk

R.A.Lyons@Swansea.ac.uk

A.M.Porter@Swansea.ac.uk

B.Diethart@Swansea.ac.uk

C.A.Thornton@Swansea.ac.uk

For peer review only

For peer review only - http://bmjopen.bmj.com/site/about/guidelines.xhtml

a.watkins@swansea.ac.uk

Anthony.Whiffen@gov.wales

h.a.snooks@swansea.ac.uk

3823/4000 words

For peer review only

ABSTRACT

Introduction

Shielding aimed to protect those predicted to be at highest risk from COVID-19 and was uniquely implemented in the United Kingdom during the COVID-19 pandemic. Clinically extremely vulnerable people identified through algorithms and screening of routine NHS data were individually and strongly advised to stay at home and strictly self-isolate even from others in their household. This study will generate a logic model of the intervention and evaluate the effects and costs of shielding to inform policy development and delivery during future pandemics.

Methods and analysis

This is a quasi-experimental study undertaken in Wales where records for people who were identified for shielding were already anonymously linked into integrated data systems for public health decision-making. We will: interview policy makers to understand rationale for shielding advice to inform analysis and interpretation of results; use anonymised individual-level data to select people identified for shielding advice in March 2020 and a matched cohort, from routine electronic health data sources, to compare outcomes; survey a stratified random sample of each group about activities and quality-of-life at 12 months; use routine and newly collected blood data to assess immunity; interview people who were identified for shielding and their carers and NHS staff who delivered healthcare during shielding, to explore compliance and experiences; collect healthcare-resource use data to calculate implementation costs and cost consequences. Our team includes people who were shielding, who used their experience to help design and deliver this study.

Ethics and dissemination

The study has received approval from the Newcastle North Tyneside 2 Research Ethics Committee (IRAS 295050). We will disseminate results directly to UK government policy makers, publish in peer reviewed journals, present at scientific and policy conferences and share accessible summaries of results online and through public and patient networks.

291/300 words

For peer review only - http://bmjopen.bmj.com/site/about/guidelines.xhtml

KEY WORDS
COVID-19

Pandemics

Shielding

Immunology

Routine Linked Data

Logic Model

For peer review only

ARTICLE SUMMARY

- This research will provide the first population-scale national assessment about effects of shielding on COVID-19 infection rate, mortality, serious illness, use of NHS resources, health-related quality-of-life and behaviour.
- In this study, we will develop a logic model for shielding, providing the first summary of the rationale for this internationally unique and untested public health intervention and underpinning interpretation and contextualisation of our study findings.
- This study will use mixed methods to understand processes, effects and costs of shielding at national and individual level, including assessment of impact of immunological status on outcomes.
- The primary limitation of the EVITE Immunity study is construction of our matched cohort; we will undertake validation checks to understand differences between groups and allow appropriate adjustments for these in our statistical analysis plan.
- The development of the EVITE Immunity study has involved people with direct experience of shielding from the outset, with public contributors represented across all aspects of the study, reflecting strong views that evidence about effects of shielding is needed.

For peer review only - http://bmjopen.bmj.com/site/about/guidelines.xhtml

INTRODUCTION

Shielding was introduced early during the COVID-19 pandemic across the United Kingdom (UK). It was intended to protect those thought to be at highest risk of serious harm should they catch COVID-19 because of pre-existing conditions such as cancer or treatment such as immunosuppressive medications. It became apparent at an early stage of the pandemic that the virus was disproportionately affecting some parts of the general population, including older people [1] and patients with pre-existing conditions such as cardio-vascular disease, respiratory disease and cancer [2,3]. A cohort study of over 17 million primary care records in England [4] confirmed the association between diagnoses such as diabetes and asthma and risk of death from COVID-19 and also highlighted the risks associated with deprivation, old age and being male and Black or South Asian.

In response to increasing transmission and deaths from COVID-19, governments across the UK nations developed methods to identify people thought to be most vulnerable to COVID-19 infection, hospital and Intensive Care Unit (ICU) admissions, serious illness or death [5-8]. These people were selected for advice to shield, before and in addition to more general lockdown measures introduced across the population. In March and April 2020, Public Health England and Public Health Wales advised individuals by letter, text or phone call to strictly self-isolate, even from people within the same home, for a period of 12-16 weeks. Support such as food parcels, prescription delivery and priority supermarket shopping slots were provided and individuals were eligible for Statutory Sick Pay [6, 7]. Shielding along with other lockdown restrictions, eased temporarily from late summer 2020 and was then reinstated shortly before Christmas 2020, with some variation by and within nation. until spring 2021.

Shielding aimed to protect those judged to be at highest risk of serious harm should they become infected with COVID-19 [5]. The mechanism for avoiding harm was to avoid infection. Clinically extremely vulnerable patients with diagnoses including cancer, serious heart conditions, respiratory problems, and receiving certain treatments such as transplants and immunosuppressant medications, were identified through algorithms and individual clinical screening methods from routine national and local NHS data sources [6, 7]. Following a refinement of the medical criteria for shielding in May 2020 [9] the shielding population increased. In England, it was estimated to be 2.2 million and in

For peer review only - http://bmjopen.bmj.com/site/about/guidelines.xhtml

Wales 133,000 people at July 2020 [10, 11]. Shielding in the UK cost £308million to deliver in the first four months [12].

Shielding is a new intervention, uniquely used in the UK during the 2020 pandemic without prior evidence of effects on health outcomes or behaviour including intended and unintended consequences [12-16]. The World Health Organization (WHO) recognises that some people are at higher risk than others from COVID-19, but states that “all must act to prevent community spread” [17]. It encouraged measures to limit transmission and protect populations to ensure that health services can sustain increased demand for patient care and treatment. Non-pharmaceutical means include physical distancing, handwashing and stay-at-home advice [17].

Evidence is now emerging of effects of shielding on: physical and mental health; wellbeing and quality-of-life including social isolation, loneliness and anxiety; access to medical care [18-21]. Higher COVID-19 rates among people who shielded are also reported [22]. There may be additional secondary effects on physical and mental health, across the shielded population or in subgroups such as the very elderly, people in different clinical condition groups and ethnic minorities. Questions remain about whether the screening process, which involved complex stratification based on modelling to account for ethnicity, deprivation and comorbidities, was the most appropriate approach [5].

There is a pressing need for rigorous population level evidence to build on early findings from the small-scale studies undertaken so far. It is well known that healthcare interventions do not always achieve intended effects [23, 24]. High quality evidence about effects of shielding advice on COVID-19 infection rate, mortality, serious illness, use of NHS resources, health related quality-of-life and behaviour is therefore urgently required to inform policy and practice throughout this and any future pandemic.

We describe the protocol for our study to evaluate the effects and costs of shielding in Wales where we will extend existing data linkage to COVID-19 diagnosis and antibody (serology) laboratory results, adopting a quasi-experimental matched cohort linked data study design to answer our research questions. As the shielding policy in Wales broadly replicated the policy in the rest of the UK, evidence

For peer review only - http://bmjopen.bmj.com/site/about/guidelines.xhtml

from this evaluation will inform policy development and delivery in England as well as the devolved nations.

Study Aim

To measure effects and costs of shielding to protect members of the general population at highest risk of serious illness or death from COVID-19 in Wales.

Objectives are to:

1. Capture the rationale for UK shielding
2. Assess effects of shielding in the general population and subgroups in terms of deaths, hospitalisations, safety and self-reported health
3. Assess the infection levels and immunity within the shielded and control populations as a whole
4. Explore behaviour, adherence, and safety concerns relating to shielding
5. Assess the costs of the shielding intervention against its consequences
6. Understand the experiences and views of health care providers in relation to the shielding intervention and perceived effects, including healthcare associated harms

METHODS AND ANALYSIS

In this study, we will use mixed methods, including analysis of anonymised individual-level population-scale electronic health record (EHR) routine data, blood samples, questionnaires, interviews and health service resource-use data to answer our aim and objectives. See table 1 for an overview of objectives, methods and outputs.

Design

Quasi-experimental evaluation

Participant Identification and participation (objectives 2-5)

Eligible participants will include those who were identified as clinically extremely vulnerable in Wales between March and May 2020 and who were advised to shield (shielded cohort). We will use individual-level population-scale anonymised data within the Secure Anonymised Information Linkage (SAIL) Databank to identify those shielded and their linked EHR records from routinely-collected NHS data sources, Office for National Statistics (ONS) data and other health and administrative data, including the Wales Multimorbidity Cohort COVID-19 extension (WMC-C19), under existing Information Governance Review Panel (IGRP) approvals [25]. We will also identify a cohort to match those identified

For peer review only - http://bmjopen.bmj.com/site/about/guidelines.xhtml

for shielding with partners in Digital Health and Care Wales (DHCW), through cohort 'propensity' matching variables including age, sex, and historic health service utilisation. We will create a stratified random sample from each of the two cohorts for questionnaire distribution and blood sample collection. Prospectively collected data from questionnaires and bloods will be linked back into the SAIL Databank for anonymised linkage. Figure 1: Data Flow Visualisation describes recruitment methods. Figure 2: Study Participant Recruitment Flow Chart describes participation.

Intervention

Individuals on the Shielded Persons List identified as clinically extremely vulnerable were sent advice, by letter (dated 24 March 2020) email or text, to stay at home for 12 weeks and '*do not go out at all'* plus to minimise contact with anyone in the same household or visiting to provide care, '*even friends and family'* [26]. Key points in this advice included:

- Not leaving the house to go to work or to see other people
- Not being in the same room as another person
- Keeping three steps away from another person in the home
- Not sharing kitchen, bathroom or bedroom facilities with others in the home
- Eating meals separately from other household members
- A possibility that hospital appointments and treatment may be postponed or cancelled
- Details of available support, including how to obtain food, prescriptions and other information

Letters were sent to clinically extremely vulnerable people throughout the pandemic which updated current advice. Correspondence after the first 12 week shielding period reflected an easing of some of the above points until January 2021 when shielding advice was reinstated. It remained, with some amendments, until August 2021 [27-30].

Methods for each objective

Objective 1) Rationale for UK shielding

We will conduct virtual interviews and focus groups with senior policy makers and clinicians from Public Health and Chief Medical Officers' teams in Wales and England. We will develop a semi-structured schedule to explore the rationale for shielding, steps undertaken to implement the intervention and individual/organisational roles. In the interviews, we will encourage participants to consider the aim of the shielding policy, components of the intervention, the way it was intended to work and any perceived risks or unintended consequences of shielding. We will show participants a

For peer review only - http://bmjopen.bmj.com/site/about/guidelines.xhtml

draft logic model prepared by the study team based on published information and invite comments. We will record and transcribe interviews, with participants' consent, and will use data to refine and agree a logic model [31, 32] to guide interpretation of study findings.

Objective 2) Effectiveness of Shielding

a) We will create a matched electronic cohort and compare demographics and clinical characteristics to understand differences between the two groups; it will not be possible to achieve a perfect match, but characterisation of differences, incorporated into our statistical analysis plan, will allow appropriate adjustments when answering our research questions. We will use anonymised individual-level linked routinely collected anonymised EHR data to compare outcomes between the two cohorts – COVID-19 infections, deaths, hospitalisations, immunity status, safety and costs up to 12 months. Inclusion of approximately 120,000 people in each cohort will give ample power to detect small differences (standardised statistical effects as small as 0.05, 90% power, 5% significance) in outcomes between groups and between most subgroups. For instance, 3-5% of each cohort will be recorded as belonging to a Black, Asian or other Minority Ethnic group, allowing comparison of outcomes between up to 6,000 people per sub-cohort; larger numbers will be included in clinical subgroups such as cancer, heart disease, diabetes. We recognise it will not be possible to completely mirror the shielded group in our matched cohort. Our statistical analysis plan will incorporate ways of characterising differences and of making appropriate adjustments when answering our research questions.

b) We will examine self-reported outcomes at 12 months. We will distribute 1,333 postal questionnaires to a stratified random sample in each of the shielded and non-shielded (matched) cohorts to achieve 533 responses in each. Questionnaires will include an online response option. In addition to the health-related quality-of-life measure (SF12) [33], questionnaires will include measures of common mental disorders, anxiety and depression (PHQ9, GAD7) [34, 35], safety concerns and behaviour during COVID-19 lockdown (staying home, isolating – including within home). Our sample size is sufficient to detect an average difference of 2.5 in SF12 component scores (standardised statistical effect 0.2, 90% power, 5% significance). Our Wales NHS partners, who hold contact details of individuals, will send the questionnaires and assign each an anonymised ID number.

For peer review only - http://bmjopen.bmj.com/site/about/guidelines.xhtml

This will ensure that the research team has no identifying information about participants at any stage of the evaluation.

Objective 3) Impact of immunological status on outcomes in the shielding population

We will analyse data from tests that have already been undertaken using anonymised population-scale linked EHR data sources including COVID-19 Polymerase Chain Reaction (PCR) data held in SAIL to make a broad assessment of immunity, immunological status, infections and antibiotic use. Immunological data will include:

- Full Blood Count (FBC) – haemoglobin, platelets, white cell count, neutrophils, lymphocytes
- Liver Function Test (LFT) – Calculated Globulin (Total protein – albumin) LIMS test code B3062 (will analyse by low and high calculated globulin)
- C-Reactive Protein RP LIMS code B3023
- Procalcitonin
- Immunoglobulins (Ig) – IgG, IgA and IgM LIMS test code B3054
- Serum electrophoresis (SEP)
- Glycated Haemoglobin (HbA1c)
- Renal Function B5373

We will also assess the availability of less frequently tested immunological data:

- Lymphocyte subsets – Cluster of differentiation (CD): CD3, CD4, CD8, CD19 and CD56
- Specific antibodies – Haemophilus Influenzae B(Hib), tetanus, pneumococcus and Severe Acute Respiratory Syndrome Coronavirus 2 (SARS-COV-2)

We will invite people approached by postal questionnaire (2b) to provide blood samples for linkage and extended analysis. We will collect Dried Blood Spots (DBS) from those who indicate consent on completed questionnaires (≥ 500 in each study arm). This self-administered test will be delivered and returned by post. We will identify a sub-group of those who provide DBS samples (50 in each study arm) and invite them to provide a liquid blood sample to investigate T cell immunological responses. The liquid blood sample will be collected by a qualified health professional in either the individual's home or community-based clinic.

Objective 4) Behaviour, adherence, and safety concerns of people who shielded

For peer review only - http://bmjopen.bmj.com/site/about/guidelines.xhtml

We will interview 40 people on the Shielded Persons List, their carers and/or household members, from those who have agreed to be contacted after completing the questionnaire (2b) and then consented to take part. These interviews will be by telephone or online (e.g. Zoom), recorded and transcribed with their consent. We will explore individual experiences during 2020, including behaviour, physical and mental health and also safety concerns (i.e. an event or situation where something went wrong or not as expected whilst receiving or trying to receive health care) [36, 37]. People with experience of shielding on our study team will co-develop the questions to be asked in these interviews. The study team will ensure interviewees reflect the range of people included in the shielding intervention in age, sex, health status, ethnic group and place of residence in Wales. We recognise that our sample may include individuals who have been bereaved and we will signpost to appropriate support.

Objective 5) Costs of the shielding intervention against its consequences

We will investigate costs of implementing the shielding policy, including costs incurred in identifying those asked to shield and in managing shielding processes. We anticipate this will include costs of:

- Developing and implementing algorithms (Public Health Wales; NHS Wales Shared Services; Digital Health and Care Wales, formerly called National Wales Informatics Service) to identify defined categories of patients
- Identifying additional patients within the NHS (via general practice registers, outpatient lists, etc.)
- Managing and sending out shielding advice letters (NHS Wales Shared Services; NHS Wales Delivery Unit)
- Sharing lists and sending out support letters to shielded people from local authorities (NHS Wales Shared Services/Delivery Unit; Unitary Authorities across Wales)
- Sharing and sending messages to shielded people from supermarkets for prioritised delivery slots (NHS Wales Shared Services/Delivery Unit/Supermarkets)
- Providing food parcel deliveries/ pharmacy prescription pick-ups and delivery (Unitary Authorities)

We will collect these data through interviews and documentary evidence from key informants at the organisations which collaborated in the delivery of this intervention. We will describe the

For peer review only - http://bmjopen.bmj.com/site/about/guidelines.xhtml

consequences of shielding in terms of healthcare resource use based on patient-level linked data extracted from SAIL (and costed using published unit costs) and COVID-19-related morbidity and mortality and summarise net monetary benefits of shielding.

Objective 6) Experiences and views of health care providers

We will interview 30 clinicians including General Practitioners (GPs), primary care and community nurses, Emergency Department (ED), emergency ambulance and intensive care staff across locations in Wales. We will use vignettes that have been developed from the interviews with the shielded population (objective 3) as a way to understand the challenges from 'the other side' in the current context, particularly patient-reported safety concerns [38]. Questions will include views on shielding, how it was implemented and how they felt it affected people including any health risks for patients. Our public contributors will co-develop these tools. Interviews will be online (e.g. Zoom/Microsoft Teams) or by telephone, recorded and transcribed with their consent.

Analysis

Objective 1) Rationale for UK shielding

We will analyse data using framework analysis. This provides a systematic approach to coding data, and provides structured outputs recommended for use in policy and health services research [39, 40]. A senior qualitative researcher (AP) will lead a team of researchers and public contributors in reading and coding data for discussion and interpretation. Through this process, we will refine the logic model for the shielding intervention and understand the intentions of applying it - making explicit the hypothesised mechanisms for change, expected outcomes and risks [31, 32]. This model will guide interpretation of study findings including mechanism and outcomes data and dissemination.

Objective 2) Effectiveness of Shielding

We will analyse quantitative data following 'intention to treat' analysis principles, with people who were identified for shielding remaining in that group whether or not they received letters or were actually following the shielding advice, and the matched cohort remaining as the comparator group. We will develop a detailed statistical analysis plan compliant with Swansea Trials Unit’s Standard Operating Procedure [41]. This will cover: descriptive summaries of study data and thematic categorisation; formal comparison of outcomes, adjusted for case mix and potential confounding

For peer review only - http://bmjopen.bmj.com/site/about/guidelines.xhtml

factors; statistical modelling strategy underpinning comparisons, including conventions for dealing with missing data, selection of confounders; and reporting of analyses.

Objective 3) Impact of immunological status on outcomes in the shielded population

The majority of people in shielded groups have had full blood counts and liver function tests in recent years. The all-Wales Results Reporting Service (WRRS) data which contains all laboratory tests on the entire population of Wales flow into the SAIL Databank and the MRC funded ConCOV population cohort (Controlling COVID-19 through enhanced population surveillance and intervention project) [8].

These widely collected data will allow an initial broad immunological analysis of humoral immunity (using calculated globulin), cellular immunity (using lymphocyte counts) and impact due to neutropenia (neutrophil counts).

To analyse immunological data we will first plot calculated globulin from low to high in g/l increments against infection, hospitalisation, death and the same analysis for lymphocytes and neutrophils 0.1, 0.2 upwards. Using primary and secondary care data we will define groups within the shielded population, into those with none, or frequent infections to assess whether prior infection frequency relates to outcomes during the pandemic. From the Dried Blood Sample, we will undertake COVID-19 antibody assays which may include testing for the receptor-binding domain (RBD) of the spike protein and for nucleocapsid. From the Liquid Blood Sample, we will undertake blood tests to determine levels of T cell immunity using interferon gamma production to a SARS-COV-2 peptide pool as the readout among participants who show no antibodies even though they have had either natural infection or vaccination [42]. Tests will be run upon the day of receipt of a sample to avoid deterioration.

Objectives 4+6) Behaviour, adherence, and safety concerns of people who shielded; Experiences and views of health care providers

We will undertake thematic analysis of interviews with people who shielded and healthcare professionals [43]. Our analysis team will include public contributors and clinical experts alongside experienced researchers. This team will review transcripts and identify themes in the data. Analysis will be informed by the logic model developed by the study team to describe the components, mechanisms and outcomes of shielding [31, 32]. We will use NVivo (qualitative data analysis software) to organise and interrogate data. Where appropriate, anonymous excerpts or quotes will be included in reports and peer-review papers.

For peer review only - http://bmjopen.bmj.com/site/about/guidelines.xhtml

Objective 5) Costs of the shielding intervention against its consequences

We will estimate healthcare resource use through anonymised linked data, compared between our shielded and non-shielded matched cohorts. ED attendances, hospital admissions, length of stay, ICU admissions and GP contacts (if available) will be accessed within the SAIL Databank and costed using published unit costs [44, 45]. A cost-consequences analysis will compare the costs of shielding (including implementation cost and changes in healthcare costs) with COVID-19-related outcomes such as morbidity, mortality and health-related quality-of-life based on SF-12 questionnaire responses [33] from a stratified random sample of people from the shielded and control cohorts. Net monetary benefit will be calculated to weigh up all costs and outcomes of the intervention.

Public involvement

People affected by the shielding policy have been directly involved throughout development of the research design. Two were co-applicants on the funding proposal and are members of the Research Management Group overseeing study implementation (LG, LD). Academic co-applicants (HS and BAE) were personally directly or indirectly affected by implementation of the shielding policy. We will recruit a Patient Advisory Panel of up to eight individuals affected by the shielding policy to supplement public input by in-depth discussion at Panel meetings and supporting LD and LG. Public contributors will be involved at all stages of study delivery including: acceptability and design of the research; review of data collection methods and tools; data interpretation and synthesis; dissemination of findings. We will recruit two additional individuals to join the independent Study Steering Committee of clinical, policy, academic, methodological and public contributor experts. We will provide honoraria, briefings and other support as needed in line with best practice and report public involvement in our outputs [46-48]. We have a named lead for public involvement in the team (BAE) who brings expertise and experience to this role.

Study management and delivery:

We will implement a comprehensive strategic and operational management, delivery and oversight infrastructure. The Research Management Group (RMG) (research staff, all co-applicants) will meet every two months. The Patient Advisory Panel (eight public contributors; chaired by a public contributor from, and reporting to, the Research Management Group) will meet quarterly. The

For peer review only - http://bmjopen.bmj.com/site/about/guidelines.xhtml

independent Study Steering Committee (clinical, policy, academic, methodological and public contributor experts) will meet three times. The Core Research Group will meet every two to four weeks throughout the study, to manage day to day activities and to ensure delivery; this group will report to the RMG.

ETHICS AND DISSEMINATION

We have gained ethical permission from the Newcastle North Tyneside 2 Research Ethics Committee (IRAS 295050). All research is conducted under the permission and approval of the SAIL independent Information Governance Review Panel (IGRP) project number 0911.

We will report findings about safety, clinical and cost-effectiveness of the 2020 shielding policy, describing costs and effects on health outcomes, health service utilisation and quality-of-life. We will describe whether the assumptions underpinning shielding were realistic, whether and how far it achieved its intended aims and behaviour and adherence in the shielded population. Our report will inform policy about public health interventions in further waves of the COVID-19 pandemic and other pandemics in the future, particularly concerning the health and safety of the most vulnerable members of society.

We will prepare a publication and engagement plan, informed by the insight and expertise of our clinical, academic, public and policy co-applicants. This will allow us to plan dissemination opportunities to reach a range of audiences and to record these.

We will disseminate results directly to policy makers through the Welsh Government COVID-19 Technical Advisory Group (TAG), and the UK government Scientific Advisory Group for Emergencies (SAGE) and its related subgroups.

We will publish our findings in peer-reviewed scientific journals and present at scientific and policy conferences (for a recent example, see https://hsruk.org/conference/conference-2021/workshops/pros-and-cons-shielding-vulnerable-people-public-health-policy given to the 2021 Health Services Research UK Conference).

Our public contributors will lead production of accessible summaries of findings which we will publish online (http://www.primecentre.wales/), share with our strong public and patient networks and promote through our social media networks.

In this first national evaluation of the effects of the UK COVID-19 shielding policy, we will contribute evidence for the role of immunity in prediction of outcome. Alongside emerging evidence from other

For peer review only - http://bmjopen.bmj.com/site/about/guidelines.xhtml

studies undertaken through the National Core Studies Immunity programme, the proposed research will support the UK in preparation for future pandemics.

REFERENCES

1. Dowd JB, Andriano L, Brazel DM, Rotondi V, Block P, Ding XJ, Liu Y, Mills MC. Demographic science aids in understanding the spread and fatality rates of COVID-19. *Proceedings of the National Academy of Sciences.* 2020*;* 117(18): 9696-9698
2. Deng G, Yin M, Chen X., Zeng, F. Clinical determinants for fatality of 44,672 patients with COVID-19. *Crit. Care*. 2020. 24
3. Docherty AB et al. Features of 16,749 hospitalised UK patients with COVID-19 using the ISARIC WHO Clinical Characterisation Protocol. *BMJ.* 2020; 369:m1985 | doi: 10.1136/bmj.m1985
4. Williamson EJ, Walker AJ, Bhaskaran K.*et al.* OpenSAFELY: factors associated with COVID-19 death in 17 million patients. *Nature*. 2020.
5. Smith G.D., Spiegelhalter D. Shielding from COVID-19 should be stratified by risk. BMJ.2020;369:m2063
6. Public Health England. Guidance on protecting people who are clinically extremely vulnerable from COVID-19. Available from: https://www.gov.uk/government/publications/guidance-on-shielding-and-protecting-extremely-vulnerable-persons-from-covid-19/guidance-on-shielding-and-protecting-extremely-vulnerable-persons-from-covid-19. Accessed: 13 July 2020
7. Welsh Government. Guidance on protecting people defined on medical grounds as clinically extremely vulnerable from coronavirus (COVID-19) – previously known as 'shielding'. Available from: https://gov.wales/guidance-protecting-people-defined-medical-grounds-clinically-extremely-vulnerable-coronavirus. Accessed: 13 July 2020
8. Controlling COVID-19 through enhanced population surveillance and intervention (ConCOV: Swansea University. Available from: https://popdatasci.swan.ac.uk/swansea-university-led-team-secures-funding-from-covid-19-rapid-response-call/. Accessed: 13 July 2020
9. Written Statement: Coronavirus (COVID-19) – Shielding Update (5 May 2020) | GOV.WALES. Accessed 2 November 2021
10. https://www.ons.gov.uk/peoplepopulationandcommunity/healthandsocialcare/conditionsanddiseases/bulletins/coronavirusandshieldingofclinicallyextremelyvulnerablepeopleinengland/9julyto16july2020 Accessed 2 November 2021
11. https://gov.wales/sites/default/files/statistics-and-research/2020-07/shielded-patient-list-in-wales-during-the-coronavirus-covid-19-pandemic-as-at-6-july-2020-830.ods Accessed 2 November 2021
12. National Audit Office. Protecting and supporting the clinically extremely vulnerable during lockdown. Report by the Comptroller and Auditor General 2021. London: Ministry of Housing, Communities & Local Government, Department of Health & Social Care

For peer review only - http://bmjopen.bmj.com/site/about/guidelines.xhtml

13. European Centre for Disease Prevention and Control (ECDC). Guidelines for the use of non-pharmaceutical measures to delay and mitigate the impact of 2019-nCoV. Stockholm: ECDC; 2020. Available from: https://www.ecdc.europa.eu/en/publications-data/guidelines-use-non-pharmaceutical-measures-delay-and-mitigate-impact-2019-ncov Accessed: 13 July 2020
14. Information from the European Centre for Disease Prevention and Control (ECDC). Coronavirus disease 2019 (COVID-19) in the EU/EEA and the UK – eighth update 8 April 2020. Available from: https://www.ecdc.europa.eu/sites/default/files/documents/covid-19-rapid-risk-assessment-coronavirus-disease-2019-eighth-update-8-april-2020.pdf Accessed: 13 July 2020
15. Evans BA, Akbari A, Bethell L et al. Response to 'Covid-19: Government's shielding scheme failed thousands of clinically extremely vulnerable people, say MPs' *BMJ* 2021; 373 doi: https://doi.org/10.1136/bmj.n1033 (Published 20 April 2021)
16. Eregie C.O. COVID-19 Pandemic and stratified shielding: Some more matters for the works *BMJ* 2020;369:m2063 doi: 10.1136/bmj.m2063 (Published 28 May 2020)
17. Hume E, Armstrong M, Manifield J, *et al*. Impact of COVID-19 shielding on physical activity and quality of life in patients with COPD. *Breathe* 2020; 16: 200231.
18. Bevan Foundation (2020) Lessons from lockdown: the experience of shielding. Available at: https://www.bevanfoundation.org/resources/experience-of-shielding/. Accessed: 07 September 2021
19. Bevan Foundation (2021) Social isolation and loneliness: the impact of shielding in Merthyr Tydfil. Available at: https://www.bevanfoundation.org/resources/social-isolation-loneliness-shielding-in-merthyr-tydfil/ Accessed: 07 September 2021
20. Kemp O., Home G.A., Soutar R. The psychological impact of COVID19 on a shielding high-risk cohort. Scottish Medical Journal 2020. Vol 65(4) 120-122
21. Davies AR, Song J, Bentley L, Akbari A, Smith T, Carter B, John G, Trigg L, Parry G, Dundon J, Cross L. (2021). *COVID-19 in Wales: The impact on levels of health care use and mental health of the clinically extremely vulnerable.* Cardiff: Public Health Wales.
22. Jani B.D., Ho F.K., Lowe D.J., Traynor J.P., MacBride-Steward S.P., Mark P.B., Mair F.S., Pell J.P. Comparison of COVID-19 outcomes among shielded and non-shielded populations. Scientific Reports (2021) 11:15278. https://doi.org/10.1038/s41598-021-94630-6
23. Oliver, K., Lorenc, T., Tinkler, J. et al. Understanding the unintended consequences of public health policies: the views of policymakers and evaluators. BMC Public Health 19, 1057 (2019). https://doi.org/10.1186/s12889-019-7389-6
24. Snooks H, Bailey-Jones K, Burge-Jones D, et al Effects and costs of implementing predictive risk stratification in primary care BMJ Quality & Safety 2019;28:697-705.
25. Lyons J, Akbari A, Torabi F, Davies G, North L, Griffiths R, Bailey R, Hollinghurst J, Fry R, Turner S, Thompson D, Rafferty J, Mizen A, Orton C, Ellwood-Thompson S, Au-Yeung L, Cross L, Gravenor M, Brophy S, Lucini B, John A, Szakmany T, Davies J, Davies C, Williams C, Emmerson C, Cottrell S, Connor T, Taylor C, Pugh R, Diggle PJ, John G, Scourfield S, Hunt J, Cunningham AM, Helliwell K, Lyons RA. Understanding and responding to COVID19 in Wales: protocol for a

For peer review only - http://bmjopen.bmj.com/site/about/guidelines.xhtml

privacy protecting data platform for enhanced epidemiology and evaluation of interventions. BMJ Open 2020;10:e043010. doi:10.1136/ bmjopen-2020-043010

26. Welsh Government (2020) Further action to protect people at highest risk from coronavirus | GOV.WALES. Accessed 29 November 2021
27. Welsh Government (2020) Written Statement: Coronavirus (COVID-19) – Shielding Update – changes to advice (1 June 2020) | GOV.WALES Accessed 29 November 2021
28. Welsh Government (2020) Written statement: An update on shielding and adopting the guidance of the Royal College of Paediatrics and Child Health (RCPCH) (16 July 2020) | GOV.WALES Accessed 29 November 2021
29. Welsh Government (2021) Written Statement: Advice to those who are Clinically Extremely Vulnerable – extension to period advice covers (29 January 2021) | GOV.WALES Accessed 29 November 2021
30. Welsh Government (2021) Guidance on protecting people defined on medical grounds as clinically extremely vulnerable from coronavirus (COVID-19) – previously known as 'shielding' [HTML] | GOV.WALES Accessed 29 November 2021
31. Mills, T., Lawton, R. & Sheard, L. Advancing complexity science in healthcare research: the logic of logic models. *BMC Med Res Methodol. 2019;* **19,** 55
32. Cochrane Infectious Diseases Group. Developing Logic Models. 2016; Effective Healthcare Consortium
33. Ware J Jr, Kosinski M, Keller SD. A 12-Item Short-Form Health Survey: construction of scales and preliminary tests of reliability and validity. *Med Care*. 1996;34(3):220-233.
34. Spitzer RL, Kroenke K, Williams JB. Validation and Utility of a Self-Report Version of PRIME-MD: The PHQ Primary Care Study. Primary Care Evaluation of Mental Disorders. Patient Health Questionnaire. *JAMA.* 1999 Nov 10;282(18):1737-44
35. Spitzer RL, Kroenke K, Williams JB. Loewe B A Brief Measure for Assessing Generalized Anxiety Disorder The GAD-7 *Arch Intern Med.* 2006;166(10):1092-1097.
36. Cooper J, Williams H, Hibbert P, Edwards A, Butt A, Wood F, Parry G, Smith P, Sheikh A, Donaldson L, Carson-Stevens A. Classification of patient-safety incidents in primary care. Bull World Health Organ. 2018 Jul 1;96(7):498-505.
37. Wright J, Lawton R, O'Hara J, et al. Improving patient safety through the involvement of patients: development and evaluation of novel interventions to engage patients in preventing patient safety incidents and protecting them against unintended harm. Southampton (UK): NIHR Journals Library 2016.
38. Sampson H, Johannessen IA. Turning on the tap: the benefits of using 'real-life' vignettes in qualitative research interviews. *Qualitative Research*. 2020;20(1):56-72. doi:10.1177/1468794118816618
39. Gale, N.K., Heath, G., Cameron, E. *et al.* Using the framework method for the analysis of qualitative data in multi-disciplinary health research. *BMC Med Res Methodol* **13,** 117 (2013). https://doi.org/10.1186/1471-2288-13-117

For peer review only - http://bmjopen.bmj.com/site/about/guidelines.xhtml

40. Srivastava, A. & Thomson, S. B. (2009). Framework Analysis: A Qualitative Methodology for Applied Research Note Policy Research. JOAAG, Vol. 4. No. 2
41. Swansea Trials Unit (2020). STU-SOP-DMS-004 – Standard Operating Procedure on Creating a Statistical Analysis Plan, Swansea University.
42. Scurr, Martin J., Wioleta M. Zelek, George Lippiatt, Michelle Somerville, Stephanie E. A. Burnell, Lorenzo Capitani, Kate Davies, et al. 2021. 'Whole Blood-Based Measurement of SARS-CoV-2-Specific T Cell Responses Reveals Asymptomatic Infection and Vaccine Efficacy in Healthy Subjects and Patients with Solid Organ Cancers'. *MedRxiv*, January, 2021.06.02.21258218. https://doi.org/10.1101/2021.06.02.21258218
43. Braun, V., & Clarke, V. (2012). Thematic analysis. In H. Cooper, P. M. Camic, D. L. Long, A. T. Panter, D. Rindskopf, & K. J. Sher (Eds.), *APA handbook of research methods in psychology, Vol. 2. Research designs: Quantitative, qualitative, neuropsychological, and biological* (pp. 57–71). American Psychological Association. https://doi.org/10.1037/13620-004]
44. Personal Social Services Research Unit (PSSRU). Unit Costs of Health and Social Care 2020. 2020 [Available from: www.pssru.ac.uk/project-pages/unit-costs/unit-costs-2020.
45. National Health Service (NHS). NHS Reference costs 2019 to 2020. 2021. (Updated: June 2021) Available at: https://www.england.nhs.uk/national-cost-collection/. Accessed: 21 July 2021.
46. Evans BA, Gallanders J, Griffiths L, Harris-Mayes R, James M, Jones S, Joseph-Williams N, Nettle M, Rolph M, Snooks H, Wallace C, Edwards A and on behalf of the SUPER group and PRIME Centre Wales Public involvement and engagement in primary and emergency care research: the story from PRIME Centre Wales IJPDS 2020 https://doi.org/10.23889/ijpds.v5i3.1363
47. UK Standards for Public Involvement. Available at https://sites.google.com/nihr.ac.uk/pi-standards/home. Accessed 2 November 2021
48. Staniszewska, S., Brett, J., Simera, I., et al. GRIPP2 reporting checklists: tools to improve reporting of patient and public involvement in research BMJ 2017; 358 doi: https://doi.org/10.1136/bmj.j3453

For peer review only

Authors' contributions:

BAE drafted the manuscript with editorial input from all authors – AA, RB, LB, SB, AC-S, LD, AE, AJ, SJ, MK, JL, RAL, AP, BS, CAT, AW, TW, HS. The research idea was conceived by HS and developed by all authors. All authors read and approved the final manuscript.

For peer review only - http://bmjopen.bmj.com/site/about/guidelines.xhtml

Acknowledgments:

This study makes use of anonymised data held in the Secure Anonymised Information Linkage (SAIL) Databank. This work uses data provided by patients and collected by the NHS as part of their care and support. We would also like to acknowledge all data providers who make anonymised data available for research. We wish to acknowledge the collaborative partnership that enabled acquisition and access to the de-identified data, which led to this output. The collaboration was led by the Swansea University Health Data Research UK team under the direction of the Welsh Government Technical Advisory Cell (TAC) and includes the following groups and organisations: the SAIL Databank, Administrative Data Research (ADR) Wales, Digital Health and Care Wales (DHCW), Public Health Wales, NHS Shared Services Partnership (NWSSP) and the Welsh Ambulance Service Trust (WAST). All research conducted has been completed under the permission and approval of the SAIL independent Information Governance Review Panel (IGRP) project number 0911.

Funding statement:

This work is supported by the National Core Studies Immunity (NCSi4P) Programme (award number UoB WT Ref: 1745068). This work was supported by the Con-COV team funded by the Medical Research Council (grant number: MR/V028367/1). This work was supported by Health Data Research UK, which receives its funding from HDR UK Ltd (HDR-9006) funded by the UK Medical Research Council, Engineering and Physical Sciences Research Council, Economic and Social Research Council, Department of Health and Social Care (England), Chief Scientist Office of the Scottish Government Health and Social Care Directorates, Health and Social Care Research and Development Division (Welsh Government), Public Health Agency (Northern Ireland), British Heart Foundation (BHF) and the Wellcome Trust. This work was supported by the ADR Wales programme of work (award number ES/S007393/1). The ADR Wales programme of work is aligned to the priority themes as identified in the Welsh Government's national strategy: Prosperity for All. ADR Wales brings together data science experts at Swansea University Medical School, staff from the Wales Institute of Social and Economic Research, Data and Methods (WISERD) at Cardiff University and specialist teams within the Welsh Government to develop new evidence which supports Prosperity for All by using the SAIL Databank at Swansea University, to link and analyse anonymised data. ADR Wales is part of the Economic and Social Research Council (part of UK Research and Innovation) funded ADR UK (grant ES/S007393/1).

For peer review only - http://bmjopen.bmj.com/site/about/guidelines.xhtml

This work was supported by the Wales COVID-19 Evidence Centre, funded by Health and Care Research Wales.

Availability of data and materials:

The data used in this study are available in the SAIL Databank at Swansea University, Swansea, UK, but as restrictions apply they are not publicly available. All proposals to use SAIL data are subject to review by an independent Information Governance Review Panel (IGRP). Before any data can be accessed, approval must be given by the IGRP. The IGRP gives careful consideration to each project to ensure proper and appropriate use of SAIL data. When access has been granted, it is gained through a privacy protecting safe haven and remote access system referred to as the SAIL Gateway. SAIL has established an application process to be followed by anyone who would like to access data via SAIL at https://www.saildatabank.com/application-process

Competing interests:

Ronan Lyons, Stephen Jolles, Ann John and Adrian Edwards are members of the Welsh Government COVID-19 Technical Advisory Group. Ann John is also co-Chair of the Scientific Pandemic Insights Group on Behaviours (SPI-B) which is a subgroup of the Scientific Advisory Group for Emergencies (SAGE) advising the UK government. Stephen Jolles is also a member of the Welsh Government Testing TAG and Cardiff University COVID Strategic Advisory Board (SAB). No other authors have competing interests.

Exclusive licence

I, the Submitting Author has the right to grant and does grant on behalf of all authors of the Work (as defined in the below author licence), an exclusive licence and/or a non-exclusive licence for contributions from authors who are: i) UK Crown employees; ii) where BMJ has agreed a CC-BY licence shall apply, and/or iii) in accordance with the terms applicable for US Federal Government officers or employees acting as part of their official duties; on a worldwide, perpetual, irrevocable, royalty-free basis to BMJ Publishing Group Ltd ("BMJ") its licensees and where the relevant Journal is co-owned by BMJ to the co-owners of the Journal, to publish the Work in BMJ Open and any other BMJ products and to exploit all rights, as set out in our licence.

The Submitting Author accepts and understands that any supply made under these terms is made by BMJ to the Submitting Author unless you are acting as an employee on behalf of your employer or a postgraduate student of an affiliated institution which is paying any applicable article publishing charge ("APC") for Open Access articles. Where the Submitting Author wishes to make the Work available on an Open Access basis (and intends to pay the relevant APC), the terms of reuse of such Open Access shall be governed by a Creative Commons licence – details of these licences and which Creative Commons licence will apply to this Work are set out in our licence referred to above.

For peer review only - http://bmjopen.bmj.com/site/about/guidelines.xhtml

Tables and figures

For peer review only

For peer review only - http://bmjopen.bmj.com/site/about/guidelines.xhtml

Table 1: Summary of methods and outputs against study objectives

Objective	Method	Output
1. Capture rationale for UK shielding.	Interviews with policy makers.	Logic model to describe components, outcomes and mechanisms of shielding.
2. Assess effects of shielding in the general population and subgroups in terms of deaths, hospitalisations, safety and self-reported health.	Comparison of anonymised routine data between shielded population and comparator group.	Comparative outcomes for shielded and matched non-shielded people:<br>COVID-19 tests, PCR-confirmed infections and deaths.<br>All-cause mortality.<br>Emergency Department attendances, emergency hospital admissions, days spent in hospital, ICU admissions and days spent in ICU.
	Questionnaire to stratified random sample of intervention and control groups.	Self-reported health related quality-of-life, anxiety, depression and loneliness.
3. Assess the infection and immunity within the shielded and control populations as a whole.	Analysis of routine records of blood tests in shielded population.<br><br>Analysis of blood samples from stratified random subsample of intervention and control groups.	Detailed analysis of COVID-19 antigen and antibody test results within the shielded and comparator populations, to understand:<br>• effects of shielding on infection and immunity across the population and for clinical subgroups including cancer.<br>• impact of immunological status on outcomes in the shielded and non-shielded population.
4. Explore behaviour, adherence, and safety concerns relating to shielding.	Interviews with 40 individuals and carers/household members who were shielded.	Experiences of shielded people and their carers or household members during the COVID-19 lockdown, including behaviour, emotional effects and safety concerns.
5. Assess costs of the shielding intervention against its consequences.	Investigation of costs and cost-consequences of managing and delivering shielding.	Costs of intervention implementation and subsequent healthcare resource use compared to consequences and outcomes, net monetary benefit.
6. Understand the experiences and views of health care providers in relation to the shielding intervention and perceived effects, including healthcare associated harms.	Interviews with 30 community-based health professionals.	Experiences of clinicians delivering care to shielded population during pandemic.

For peer review only - http://bmjopen.bmj.com/site/about/guidelines.xhtml

Figure 1: Data Flow Visualisation

Figure 2: Study Participant Recruitment Flow Chart

For peer review only

For peer review only - http://bmjopen.bmj.com/site/about/guidelines.xhtml

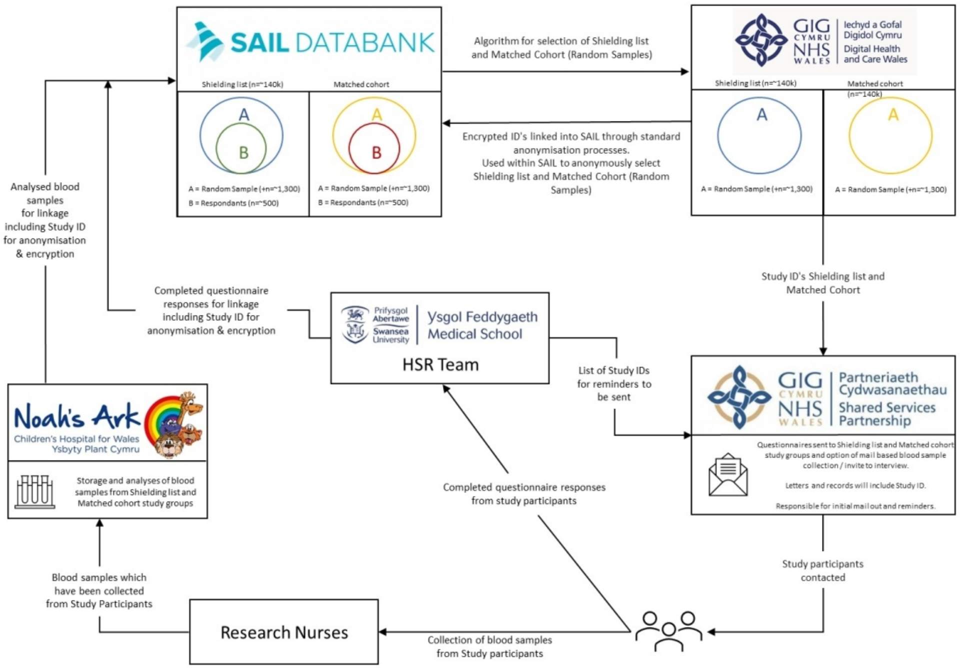

Figure 1: Data Flow Visualisation

131x90mm (300 x 300 DPI)

EVITE study participant recruitment flowchart

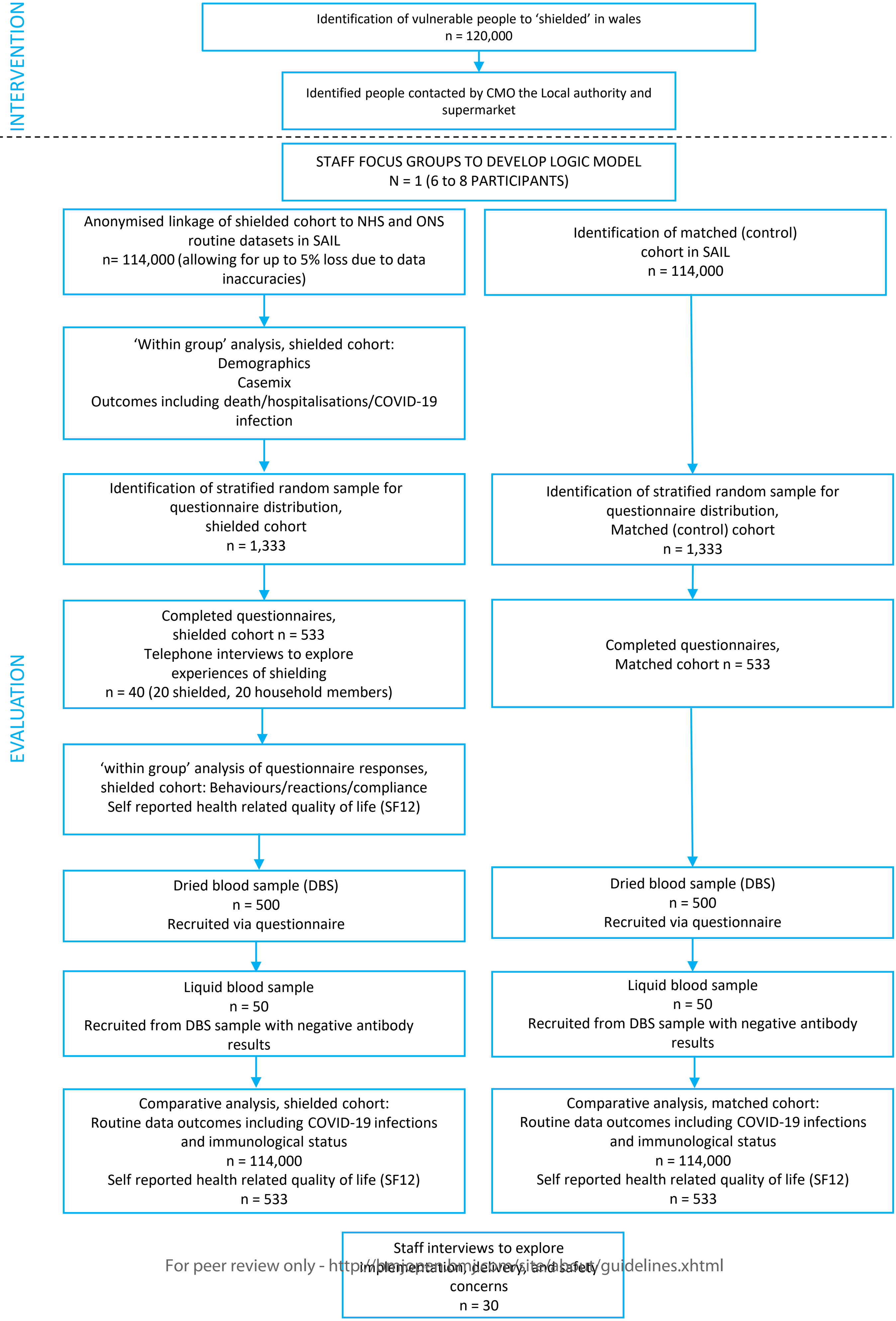

TREND Statement Checklist

Paper Section/ Topic	Item No	Descriptor	Reported? ✓	Pg #
**Title and Abstract**				
Title and Abstract	1	• Information on how unit were allocated to interventions	√	4
		• Structured abstract recommended	√	4
		• Information on target population or study sample	√	4
**Introduction**				
Background	2	• Scientific background and explanation of rationale	√	6-7
		• Theories used in designing behavioral interventions	√	6-7
**Methods**				
Participants	3	• Eligibility criteria for participants, including criteria at different levels in recruitment/sampling plan (e.g., cities, clinics, subjects)	√	8-13
		• Method of recruitment (e.g., referral, self-selection), including the sampling method if a systematic sampling plan was implemented	√	8-13
		• Recruitment setting	√	8-13
		• Settings and locations where the data were collected	√	8-13
Interventions	4	• Details of the interventions intended for each study condition and how and when they were actually administered, specifically including:	√	9
		○ Content: what was given?	√	9
		○ Delivery method: how was the content given?	√	9
		○ Unit of delivery: how were the subjects grouped during delivery?	√	9
		○ Deliverer: who delivered the intervention?	√	9
		○ Setting: where was the intervention delivered?	√	9
		○ Exposure quantity and duration: how many sessions or episodes or events were intended to be delivered? How long were they intended to last?	√	9
		○ Time span: how long was it intended to take to deliver the intervention to each unit?	√	9
		○ Activities to increase compliance or adherence (e.g., incentives)	√	6 + 9
Objectives	5	• Specific objectives and hypotheses	√	8
Outcomes	6	• Clearly defined primary and secondary outcome measures	√	10-13
		• Methods used to collect data and any methods used to enhance the quality of measurements	√	8-13
		• Information on validated instruments such as psychometric and biometric properties	√	10
Sample Size	7	• How sample size was determined and, when applicable, explanation of any interim analyses and stopping rules	√	10
Assignment Method	8	• Unit of assignment (the unit being assigned to study condition, e.g., individual, group, community)	√	10
		• Method used to assign units to study conditions, including details of any restriction (e.g., blocking, stratification, minimization)	√	10
		• Inclusion of aspects employed to help minimize potential bias induced due to non-randomization (e.g., matching)	√	10

For peer review only

Blinding (masking)	9	• Whether or not participants, those administering the interventions, and those assessing the outcomes were blinded to study condition assignment; if so, statement regarding how the blinding was accomplished and how it was assessed.	n/a	n/a
Unit of Analysis	10	• Description of the smallest unit that is being analyzed to assess intervention effects (e.g., individual, group, or community)	√	10
		• If the unit of analysis differs from the unit of assignment, the analytical method used to account for this (e.g., adjusting the standard error estimates by the design effect or using multilevel analysis)	n/a	n/a
Statistical Methods	11	• Statistical methods used to compare study groups for primary methods outcome(s), including complex methods of correlated data	√	13-14
		• Statistical methods used for additional analyses, such as a subgroup analyses and adjusted analysis	√	13-15
		• Methods for imputing missing data, if used	√	13
		• Statistical software or programs used	√	13-14
Results √				
Participant flow	12	• Flow of participants through each stage of the study: enrollment, assignment, allocation, and intervention exposure, follow-up, analysis (a diagram is strongly recommended)	√	25
		○ Enrollment: the numbers of participants screened for eligibility, found to be eligible or not eligible, declined to be enrolled, and enrolled in the study	n/a	n/a
		○ Assignment: the numbers of participants assigned to a study condition	√	10
		○ Allocation and intervention exposure: the number of participants assigned to each study condition and the number of participants who received each intervention	√	10
		○ Follow-up: the number of participants who completed the follow-up or did not complete the follow-up (i.e., lost to follow-up), by study condition	√	13
		○ Analysis: the number of participants included in or excluded from the main analysis, by study condition	√	13
		• Description of protocol deviations from study as planned, along with reasons	n/a	n/a
Recruitment	13	• Dates defining the periods of recruitment and follow-up	√	10
Baseline Data	14	• Baseline demographic and clinical characteristics of participants in each study condition	√	10-14
		• Baseline characteristics for each study condition relevant to specific disease prevention research	√	10-14
		• Baseline comparisons of those lost to follow-up and those retained, overall and by study condition	√	10-14
		• Comparison between study population at baseline and target population of interest	√	10-14
Baseline equivalence	15	• Data on study group equivalence at baseline and statistical methods used to control for baseline differences	√	10
Numbers analyzed	16	• Number of participants (denominator) included in each analysis for each study condition, particularly when the denominators change for different outcomes; statement of the results in absolute numbers when feasible	√	10
		• Indication of whether the analysis strategy was "intention to treat" or, if not, description of how non-compliers were treated in the analyses	√	13

For peer review only

Outcomes and estimation	17	• For each primary and secondary outcome, a summary of results for each estimation study condition, and the estimated effect size and a confidence interval to indicate the precision	√	9-15
		• Inclusion of null and negative findings	√	9-15
		• Inclusion of results from testing pre-specified causal pathways through which the intervention was intended to operate, if any	√	9-15
Ancillary analyses	18	• Summary of other analyses performed, including subgroup or restricted analyses, indicating which are pre-specified or exploratory	√	9-15
Adverse events	19	• Summary of all important adverse events or unintended effects in each study condition (including summary measures, effect size estimates, and confidence intervals)	√	9-15
DISCUSSION				
Interpretation	20	• Interpretation of the results, taking into account study hypotheses, sources of potential bias, imprecision of measures, multiplicative analyses, and other limitations or weaknesses of the study	√	9-15
		• Discussion of results taking into account the mechanism by which the intervention was intended to work (causal pathways) or alternative mechanisms or explanations	√	9-15
		• Discussion of the success of and barriers to implementing the intervention, fidelity of implementation	√	9-15
		• Discussion of research, programmatic, or policy implications	√	7-8, 16
Generalizability	21	• Generalizability (external validity) of the trial findings, taking into account the study population, the characteristics of the intervention, length of follow-up, incentives, compliance rates, specific sites/settings involved in the study, and other contextual issues	√	7-8
Overall Evidence	22	• General interpretation of the results in the context of current evidence and current theory	√	16

From: Des Jarlais, D. C., Lyles, C., Crepaz, N., & the Trend Group (2004). Improving the reporting quality of nonrandomized evaluations of behavioral and public health interventions: The TREND statement. *American Journal of Public Health*, 94, 361-366. For more information, visit: http://www.cdc.gov/trendstatement/

For peer review only

BMJ Open

Evaluation of the Shielding Initiative in Wales (EVITE Immunity):Protocol for a quasi-experimental study

Journal:	*BMJ Open*
Manuscript ID	bmjopen-2021-059813.R1
Article Type:	Protocol
Date Submitted by the Author:	31-May-2022
Complete List of Authors:	Evans, Bridie; Swansea University Medical School; Swansea University Medical School, PRIME Centre Wales<br>Akbari, Ashley; Swansea University Medical School<br>Bailey, Rowena; Swansea University Medical School<br>Bethell, Lesley; Swansea University Medical School, public contributor<br>Bufton, Samantha; Welsh Government<br>Carson-Stevens, Andrew; Cardiff University<br>Dixon, Lucy; Swansea University Medical School, public contributor<br>Edwards, Adrian; Cardiff University<br>John, Ann; Swansea University Medical School<br>Jolles, Stephen; University Hospital of Wales<br>Kingston, Mark; Swansea University Medical School; Swansea University Medical School, PRIME Centre Wales<br>Lyons, Jane; Swansea University Medical School<br>Lyons, Ronan; Swansea University Medical School<br>Porter, Alison; Swansea University Medical School; Swansea University Medical School, PRIME Centre Wales<br>Sewell, Bernadette; Swansea University, Swansea Centre for Health Economics<br>Watkins, Alan; Swansea University Medical School<br>Whiffen, Tony; Welsh Government<br>Snooks, Helen; Swansea University Medical School; Swansea University Medical School, PRIME Centre Wales
**Primary Subject Heading**:	Health policy
Secondary Subject Heading:	Public health, Immunology (including allergy)
Keywords:	COVID-19, IMMUNOLOGY, PUBLIC HEALTH, Health policy < HEALTH SERVICES ADMINISTRATION & MANAGEMENT

SCHOLARONE™
Manuscripts

Evaluation of the Shielding Initiative in Wales (EVITE Immunity): Protocol for a quasi-experimental study

Authors:

Bridie Angela Evans [1,2*]

Ashley Akbari [1,3]

Rowena Bailey [1,3]

Lesley Bethell [4]

Samantha Bufton [5]

Andrew Carson-Stevens [6]

Lucy Dixon [4]

Adrian Edwards [6]

Ann John [1,3]

Stephen Jolles [7]

Mark Kingston [1, 2]

Jane Lyons [1,3]

Ronan A Lyons [1,3]

Alison Porter [1,2]

Bernadette Sewell [8]

Catherine A Thornton [1]

Alan Watkins [1]

Anthony Whiffen [5]

Helen Snooks [1,2]

Affiliations:

[1] Swansea University Medical School, Swansea, UK

[2] PRIME Centre Wales, Swansea University Medical School, Swansea, UK

[3] Population Data Science, Health Data Research UK, Swansea University, Swansea, UK

[4] Public contributor, c/o Swansea University Medical School, Swansea, UK

[5] Administrative Data Research Unit, Welsh Government, Cardiff, UK

For peer review only

For peer review only - http://bmjopen.bmj.com/site/about/guidelines.xhtml

[6] PRIME Centre Wales, Division of Population Medicine, School of Medicine, Cardiff University, Cardiff, UK

[7] Immunodeficiency Centre for Wales, University Hospital of Wales, Cardiff, UK

[8] Swansea Centre for Health Economics, Swansea University, Swansea, UK

***Corresponding author:**

Bridie Angela Evans

Institute of Life Science

Swansea University Medical School

Singleton Campus

Swansea SA2 8PP

Authors' email addresses:

b.a.evans@swansea.ac.uk

A.Akbari@Swansea.ac.uk

R.Bailey@Swansea.ac.uk

lesleybethell@gmail.com

Samantha.Bufton@gov.wales

andypcs@gmail.com

lucydixon40@gmail.com

EdwardsAG@cardiff.ac.uk

A.John@Swansea.ac.uk

JollesSR@cardiff.ac.uk

M.R.Kingston@Swansea.ac.uk

J.Lyons@Swansea.ac.uk

R.A.Lyons@Swansea.ac.uk

A.M.Porter@Swansea.ac.uk

B.Diethart@Swansea.ac.uk

C.A.Thornton@Swansea.ac.uk

For peer review only

For peer review only - http://bmjopen.bmj.com/site/about/guidelines.xhtml

a.watkins@swansea.ac.uk

Anthony.Whiffen@gov.wales

h.a.snooks@swansea.ac.uk

3996/4000 words

For peer review only

For peer review only - http://bmjopen.bmj.com/site/about/guidelines.xhtml

ABSTRACT

Introduction

Shielding aimed to protect those predicted to be at highest risk from COVID-19 and was uniquely implemented in the United Kingdom during the COVID-19 pandemic. Clinically extremely vulnerable people identified through algorithms and screening of routine NHS data were individually and strongly advised to stay at home and strictly self-isolate even from others in their household. This study will generate a logic model of the intervention and evaluate the effects and costs of shielding to inform policy development and delivery during future pandemics.

Methods and analysis

This is a quasi-experimental study undertaken in Wales where records for people who were identified for shielding were already anonymously linked into integrated data systems for public health decision-making. We will: interview policy makers to understand rationale for shielding advice to inform analysis and interpretation of results; use anonymised individual-level data to select people identified for shielding advice in March 2020 and a matched cohort, from routine electronic health data sources, to compare outcomes; survey a stratified random sample of each group about activities and quality-of-life at 12 months; use routine and newly collected blood data to assess immunity; interview people who were identified for shielding and their carers and NHS staff who delivered healthcare during shielding, to explore compliance and experiences; collect healthcare-resource use data to calculate implementation costs and cost consequences. Our team includes people who were shielding, who used their experience to help design and deliver this study.

Ethics and dissemination

The study has received approval from the Newcastle North Tyneside 2 Research Ethics Committee (IRAS 295050). We will disseminate results directly to UK government policy makers, publish in peer reviewed journals, present at scientific and policy conferences and share accessible summaries of results online and through public and patient networks.

291/300 words

For peer review only

KEY WORDS

COVID-19

Pandemics

Shielding

Immunology

Routine Linked Data

Logic Model

For peer review only

ARTICLE SUMMARY

- This research will provide the first population-scale national assessment about effects of shielding on COVID-19 infection rate, mortality, serious illness, use of NHS resources, health-related quality-of-life and behaviour.
- In this study, we will develop a logic model for shielding, providing the first summary of the rationale for this internationally unique and untested public health intervention and underpinning interpretation and contextualisation of our study findings.
- This study will use mixed methods to understand processes, effects and costs of shielding at national and individual level, including assessment of impact of immunological status on outcomes.
- The primary limitation of the EVITE Immunity study is construction of our matched cohort; we will undertake validation checks to understand differences between groups and allow appropriate adjustments for these in our statistical analysis plan.
- The development of the EVITE Immunity study has involved people with direct experience of shielding from the outset, with public contributors represented across all aspects of the study, reflecting strong views that evidence about effects of shielding is needed.

For peer review only - http://bmjopen.bmj.com/site/about/guidelines.xhtml

INTRODUCTION

Shielding was introduced early during the COVID-19 pandemic across the United Kingdom (UK). It was intended to protect those thought to be at highest risk of serious harm should they catch COVID-19 because of pre-existing conditions such as cancer or treatment such as immunosuppressive medications. It became apparent at an early stage of the pandemic that the virus was disproportionately affecting some parts of the general population, including older people [1] and patients with pre-existing conditions such as cardio-vascular disease, respiratory disease and cancer [2,3]. A cohort study of over 17 million primary care records in England [4] confirmed the association between diagnoses such as diabetes and asthma and risk of death from COVID-19 and also highlighted the risks associated with deprivation, old age and being male and Black or South Asian.

In response to increasing transmission and deaths from COVID-19, governments across the UK nations developed methods to identify people thought to be most vulnerable to COVID-19 infection, hospital and Intensive Care Unit (ICU) admissions, serious illness or death [5-8]. These people were selected for advice to shield, before and in addition to more general lockdown measures introduced across the population. In March and April 2020, Public Health England and Public Health Wales advised individuals by letter, text or phone call to strictly self-isolate, even from people within the same home, for a period of 12-16 weeks. Support such as food parcels, prescription delivery and priority supermarket shopping slots were provided and individuals were eligible for Statutory Sick Pay [6, 7]. Shielding along with other lockdown restrictions, eased temporarily from late summer 2020 and was then reinstated shortly before Christmas 2020, with some variation by and within nation. until spring 2021.

Shielding aimed to protect those judged to be at highest risk of serious harm should they become infected with COVID-19 [5]. The mechanism for avoiding harm was to avoid infection. Clinically extremely vulnerable (CEV) patients with diagnoses including cancer, serious heart conditions, respiratory problems, and receiving certain treatments such as transplants and immunosuppressant medications, were identified through algorithms and individual clinical screening methods from routine national and local NHS data sources [6, 7, 8]. Following a refinement of the medical criteria for shielding in May 2020 [9] the shielding population increased. In England, it was estimated to be 2.2

For peer review only - http://bmjopen.bmj.com/site/about/guidelines.xhtml

million and in Wales 133,000 people at July 2020 [10, 11]. Shielding in the UK cost £308million to deliver in the first four months [12].

Shielding is a new intervention, uniquely used in the UK during the 2020 pandemic without prior evidence of effects on health outcomes or behaviour including intended and unintended consequences [12-16]. The World Health Organization (WHO) recognises that some people are at higher risk than others from COVID-19, but states that “all must act to prevent community spread” [17]. It encouraged measures - including physical distancing, handwashing and stay-at-home advice - to limit transmission and protect populations to ensure that health services can sustain increased demand for patient care and treatment.

Evidence is now emerging of effects of shielding on: physical and mental health; wellbeing and quality-of-life including social isolation, loneliness and anxiety; access to medical care [18-21]. Higher COVID-19 rates among people who shielded are also reported [22, 23]. There may be additional secondary effects on physical and mental health, across the shielded population or in subgroups such as the very elderly, people in different clinical condition groups and ethnic minorities. Questions remain about whether the screening process, which involved complex stratification based on modelling to account for ethnicity, deprivation and comorbidities, was the most appropriate approach [5].

There is a pressing need for rigorous population level evidence to build on early findings from the small-scale studies undertaken so far. It is well known that healthcare interventions do not always achieve intended effects [24, 25]. High quality evidence about effects of shielding advice on COVID-19 infection rate, mortality, serious illness, use of NHS resources, health related quality-of-life and behaviour is therefore urgently required to inform policy and practice throughout this and any future pandemic.

We describe our protocol to evaluate the effects and costs of shielding in Wales where we will extend existing data linkage to COVID-19 diagnosis and antibody (serology) laboratory results, adopting a quasi-experimental matched cohort linked data study design to answer our research questions. As the

For peer review only
For peer review only - http://bmjopen.bmj.com/site/about/guidelines.xhtml

shielding policy in Wales broadly replicated the policy in the rest of the UK, evidence from this evaluation will inform policy development and delivery in England as well as the devolved nations.

Study Aim

To measure effects and costs of shielding to protect members of the general population at highest risk of serious illness or death from COVID-19 in Wales.

Objectives are to:

1. Capture the rationale for UK shielding
2. Assess effects of shielding in the general population and subgroups in terms of deaths, hospitalisations, safety and self-reported health
3. Assess the infection levels and immunity within the shielded and control populations as a whole
4. Explore behaviour, adherence, and safety concerns relating to shielding
5. Assess the costs of the shielding intervention against its consequences
6. Understand the experiences and views of health care providers in relation to the shielding intervention and perceived effects, including healthcare associated harms

METHODS AND ANALYSIS

See table 1 for an overview of objectives, methods and outputs.

Design

Quasi-experimental evaluation

Participant Identification and participation (objectives 2-5)

Eligible participants will include those identified as CEV in Wales between March and May 2020 and who were advised to shield (shielded cohort). We will use individual-level population-scale anonymised data within the Secure Anonymised Information Linkage (SAIL) Databank to identify those shielded and their linked EHR records from routinely-collected NHS data sources, Office for National Statistics (ONS) data and other health and administrative data, including the Wales Multimorbidity Cohort COVID-19 extension (WMC-C19), under existing Information Governance Review Panel (IGRP) approvals [26]. We will also identify a cohort to match those identified for shielding with partners in Digital Health and Care Wales (DHCW), through cohort 'propensity' matching variables including age, sex, and historic health service utilisation. We will create a stratified random sample from each of the two cohorts for questionnaire distribution and blood sample collection. Prospectively collected data from

eview only

questionnaires and bloods will be linked back into the SAIL Databank for anonymised linkage. Figure 1: Data Flow Visualisation describes recruitment methods. Figure 2: Study Participant Recruitment Flow Chart describes participation.

Intervention

Individuals on the Shielded Persons List identified as CEV were sent advice (table 2), by letter (dated 24 March 2020) email or text, to stay at home for 12 weeks and '*do not go out at all'* plus to minimise contact with anyone in the same household or visiting to provide care, '*even friends and family*' [27]. Correspondence after the first 12 week shielding period reflected an easing, then reinstatement, then more easing [28-31].

Methods for each objective

Objective 1) Rationale for UK shielding

We will conduct virtual interviews and focus groups with senior policy makers and clinicians from Public Health and Chief Medical Officers' teams in Wales and England. We will encourage participants to consider the aim of the shielding policy, components of the intervention, the way it was intended to work and any perceived risks or unintended consequences of shielding. We will show participants a draft logic model, including: components of the intervention; mechanisms for change (how the intervention was expected to work); expected outcomes and impacts, including harms. The draft logic model was prepared by the study team based on published information and invite comments. We will record and transcribe interviews, with participants' consent, and will use data to refine and agree a logic model [32, 33] to guide interpretation of study findings.

Objective 2) Effectiveness of Shielding

We will create a matched electronic cohort and compare demographics and clinical characteristics to understand differences between the two groups; it will not be possible to achieve a perfect match, but characterisation of differences, incorporated into our statistical analysis plan, will allow appropriate adjustments when answering our research questions. We know that those warranting shielding will have higher utilisation rates. The rationale for matching on health care utilisation is to identify people who should have been shielding (but were missed due to initial selection of conditions based on prioritisation for flu vaccination and/or administrative error) who can be matched on propensity, to create as similar as possible a comparator group (in the absence of randomisation) and

For peer review only - http://bmjopen.bmj.com/site/about/guidelines.xhtml

utilise a difference in difference approach to estimate effectiveness, comparing pre- and post-intervention health service utilisation rates.

a) We will use anonymised individual-level linked routinely collected anonymised EHR data to compare outcomes between the two cohorts – COVID-19 infections, deaths, hospitalisations, immunity status, safety and costs up to 12 months. Inclusion of approximately 120,000 people in each cohort - from date of their addition to the shielding list between March 23rd - December 31st 2020; and from March 23rd 2020 for the matched control groups: with follow-up of outcomes up to 1 year - gives ample power to detect small differences (standardised statistical effects as small as 0.05, 90% power, 5% significance) in outcomes between groups and between most subgroups. For instance, 3-5% of each cohort will be recorded as belonging to a Black, Asian or other Minority Ethnic group, allowing comparison of outcomes between up to 6,000 people per sub-cohort; larger numbers will be included in clinical subgroups such as cancer, heart disease, diabetes. We recognise it will not be possible to completely mirror the shielded group in our matched cohort. Our statistical analysis plan will incorporate ways of characterising differences and making appropriate adjustments.

b) We will examine self-reported outcomes at 12 months. We will distribute 1,333 postal questionnaires (with online response option) (appendix 1) to a stratified random sample in each of the shielded and non-shielded (matched) cohorts to achieve 533 responses in each. Questionnaires will include: the health-related quality-of-life measure (SF12) [34]; measures of common mental disorders, anxiety and depression (PHQ9, GAD7) [35, 36]; safety concerns and behaviour during COVID-19 lockdown (staying home, isolating – including within home). Our sample size is sufficient to detect an average difference of 2.5 in SF12 component scores (standardised statistical effect 0.2, 90% power, 5% significance). Our Wales NHS partners, who hold contact details of individuals, will send the questionnaires and assign each an anonymised ID number. This will ensure that the research team has no identifying information about participants at any stage of the evaluation.

Objective 3) Impact of immunological status on outcomes in the shielding population

We will analyse data from tests already undertaken using anonymised population-scale linked EHR data sources including COVID-19 Polymerase Chain Reaction (PCR) data held in SAIL to make a broad assessment of immunity, immunological status, infections and antibiotic use. Immunological data will include:

For peer review only - http://bmjopen.bmj.com/site/about/guidelines.xhtml

- Full Blood Count (FBC) – haemoglobin, platelets, white cell count, neutrophils, lymphocytes
- Liver Function Test (LFT) – Calculated Globulin (Total protein – albumin) LIMS test code B3062 (analysis by low and high calculated globulin)
- C-Reactive Protein RP LIMS code B3023
- Procalcitonin
- Immunoglobulins (Ig) – IgG, IgA and IgM LIMS test code B3054
- Serum electrophoresis (SEP)
- Glycated Haemoglobin (HbA1c)
- Renal Function B5373

We will also assess availability of less frequently tested immunological data:

- Lymphocyte subsets – Cluster of differentiation (CD): CD3, CD4, CD8, CD19 and CD56
- Specific antibodies – Haemophilus Influenzae B(Hib), tetanus, pneumococcus and Severe Acute Respiratory Syndrome Coronavirus 2 (SARS-COV-2)

We will invite people approached by postal questionnaire (2b) to provide blood samples for linkage and extended analysis. We will collect Dried Blood Spots (DBS) from those who indicate consent on completed questionnaires (≥ 500 in each study arm). This self-administered test will be delivered and returned by post. We will identify a sub-group (50 in each study arm) and invite them to provide a liquid blood sample to investigate T cell immunological responses. The liquid blood sample will be collected by a qualified health professional in the individual's home.

Objective 4) Behaviour, adherence, and safety concerns of people who shielded

We will interview 40 people on the Shielded Persons List, their carers and/or household members, from those who agree to be contacted after completing the questionnaire (2b) and then consent to take part. Interviews will be by telephone or online (e.g. Zoom), recorded and transcribed with their consent. We will explore individual experiences during 2020, including behaviour, physical and mental health and also safety concerns (i.e. an event or situation where something went wrong or not as expected whilst receiving or trying to receive health care) [37, 38]. People with experience of shielding on our study team will co-develop the interview questions (appendix 2). The study team will ensure interviewees reflect the range of people included in the shielding intervention in age, sex, health status, ethnic group and place of residence in Wales. We recognise that our sample may include individuals who have been bereaved and we will signpost to appropriate support.

For peer review only - http://bmjopen.bmj.com/site/about/guidelines.xhtml

Objective 5) Costs of the shielding intervention against its consequences

We will investigate implementation costs, including costs incurred in identifying those asked to shield and in managing shielding processes. We anticipate this will include costs of:

- Developing and implementing algorithms (Public Health Wales; NHS Wales Shared Services; Digital Health and Care Wales, formerly called National Wales Informatics Service) to identify defined categories of patients
- Identifying additional patients within the NHS (via general practice registers, outpatient lists, etc.)
- Managing and sending out shielding advice letters (NHS Wales Shared Services; NHS Wales Delivery Unit)
- Sharing lists and sending out support letters to shielded people from local authorities (NHS Wales Shared Services/Delivery Unit; Unitary Authorities across Wales)
- Sharing and sending messages to shielded people from supermarkets for prioritised delivery slots (NHS Wales Shared Services/Delivery Unit/Supermarkets)
- Providing food parcel deliveries/ pharmacy prescription pick-ups and delivery (Unitary Authorities)

We will collect these data through interviews and documentary evidence from key informants at the organisations which collaborated in delivering this intervention. We will describe the consequences of shielding in terms of healthcare resource use based on patient-level linked data extracted from SAIL (and costed using published unit costs) and COVID-19-related morbidity and mortality and summarise net monetary benefits of shielding.

Objective 6) Experiences and views of health care providers

We will interview 30 clinicians including General Practitioners (GPs), primary care and community nurses, Emergency Department (ED), emergency ambulance and intensive care staff across locations in Wales. We will use vignettes developed from the interviews with the shielded population (objective 3) to understand challenges, particularly patient-reported safety concerns [39]. Questions will include views on shielding, how it was implemented and how they felt it affected people including any health

For peer review only

risks for patients (appendix 3). Our public contributors will co-develop these tools. Interviews will be online or by telephone, recorded and transcribed with their consent.

Analysis

Objective 1) Rationale for UK shielding

We will analyse data using framework analysis, recommended for use in policy and health services research [40, 41]. A senior qualitative researcher (AP) will lead a team of researchers and public contributors in reading and coding data for discussion and interpretation. Through this process, we will refine the logic model for the shielding intervention and understand the intentions of applying it - making explicit the hypothesised mechanisms for change, expected outcomes and risks [32, 33]. This model will guide interpretation of study findings including mechanism and outcomes data and dissemination.

Objective 2) Effectiveness of Shielding

We will analyse quantitative data following 'intention to treat' analysis principles. Our detailed statistical analysis plan, compliant with Swansea Trials Unit's Standard Operating Procedure [42], will cover: descriptive summaries of study data and thematic categorisation; formal comparison of outcomes, adjusted for case mix and potential confounding factors; statistical modelling strategy underpinning comparisons, including conventions for dealing with missing data, selection of confounders; reporting of analyses. Modelling will use generalised linear and survival multilevel models for events, counts and time to events. Entry dates are based on the date identified as CEV (shielded cohort) or 20 March 2020 (matched cohort); 12-month follow-up data will be censored by death, or known date of migration from Wales.

There was considerable spatial as well as temporal variation in the (estimated) R number across Wales during our study window, with little detailed data on the accuracy of these estimates. Although difficult to justify incorporation of such estimates into formal models, we will use what is known to inform discussion of our results. We will assess if available anonymised residential identifiers allow creation of usable household/residential clusters, and, if so, whether extension to include clustering improves our models. We will consider further use of Care Home residence identifiers and Critical Care and Hospital in-patient spells in defining potential explanatory covariates and factors for inclusion in models.

For peer review only - http://bmjopen.bmj.com/site/about/guidelines.xhtml

Objective 3) Impact of immunological status on outcomes in the shielded population

The majority of people in shielded groups had full blood counts and liver function tests in recent years. The all-Wales Results Reporting Service (WRRS) data which contains all laboratory tests on the entire population of Wales flow into the SAIL Databank and the MRC funded ConCOV population cohort (Controlling COVID-19 through enhanced population surveillance and intervention project) [8].

These widely collected data will allow an initial broad immunological analysis of humoral immunity (using calculated globulin), cellular immunity (using lymphocyte counts) and impact due to neutropenia (neutrophil counts).

To analyse immunological data we will first plot calculated globulin from low to high in g/l increments against infection, hospitalisation, death and the same analysis for lymphocytes and neutrophils 0.1, 0.2 upwards. Using primary and secondary care data we will define groups within the shielded population, into those with none, or frequent infections to assess whether prior infection frequency relates to outcomes during the pandemic. From the Dried Blood Sample, we will undertake COVID-19 antibody assays which may include testing for the receptor-binding domain (RBD) of the spike protein and for nucleocapsid. We will measure T cell responses to SARS-CoV-2 using a commercially available whole-blood assay (ImmunoServ Ltd) [43]. Briefly, this means 10ml venous blood samples are collected into sodium heparin vacutainers (BD) and stimulated with a SARS-CoV-2 peptide pool containing peptides spanning the entire spike (S1 and S2) protein, nucleocapsid phosphoprotein and membrane glycoprotein for 20-24 hours at 37°C prior to a 2 min centrifugation at 3000 g. The plasma from the top of each blood sample will be harvested and analysed for interferon gamma by ELISA. This will determine levels of T cell immunity among participants who show no antibodies to COVID-19, even though they have had either natural COVID-19 infection or vaccination to COVID-19. A positive SARS-CoV-2-specific T cell response will be defined as >23.55pg/ml IFNg and 50% above the negative (unstimulated) control value, as previously determined in healthy donors [43]. Tests will be run upon the day of sample receipt to avoid deterioration.

Objectives 4+6) Behaviour, adherence, and safety concerns of people who shielded; Experiences and views of health care providers

For peer review only - http://bmjopen.bmj.com/site/about/guidelines.xhtml

We will undertake thematic analysis of interviews with people who shielded and healthcare professionals [44]. Our analysis team will include public contributors and clinical experts alongside experienced researchers. Analysis will be informed by the logic model (objective 1) [32, 33]. Where appropriate, anonymous excerpts will be included in reports and peer-review papers.

Objective 5) Costs of the shielding intervention against its consequences

We will estimate NHS healthcare resource use through anonymised linked data, compared between shielded and non-shielded matched cohorts. ED attendances, hospital admissions, length of stay, ICU admissions and GP contacts (if available) will be accessed within the SAIL Databank and costed using published unit costs [45, 46]. Cost-consequences analysis will compare costs of shielding (including implementation cost and changes in healthcare costs) with COVID-19-related outcomes such as morbidity, mortality and health-related quality-of-life based on SF-12 questionnaire responses [34] from a stratified random sample of people from shielded and control cohorts. Net monetary benefit will be calculated to weigh up all costs and outcomes of the intervention.

Study design limitations

As the shielding intervention was introduced across all the UK at one timepoint, we are able to carry out a quasi-experimental study only, with no clear historic or concurrent control group. In this circumstance, we acknowledge that any method to identify a matched comparator group is, to some extent, flawed. As entire clinical codes were allocated to the CEV (shielded) group, we intend to match as well as we can, from routine data sources, by age, sex and health service utilisation in the year prior to introduction of the shielding policy. We will then compare clinical, demographic and socio-economic characteristics of our two groups and adjust for differences in our analysis. Without any group for comparison of outcomes it is difficult to draw any conclusions related to the benefits, harms and costs of the shielding policy. We have therefore selected this study design as the best available for this study.

The study was designed before widespread availability of vaccinations. We do not intend to include this variable in our data collection and analyses; so many variations in timing, vaccination delivered, number of vaccinations and boosters means this analysis is outside the scope of the current study.

For peer review only - http://bmjopen.bmj.com/site/about/guidelines.xhtml

Although not all those included in the shielding intervention will have received letters or complied with advice, and some people in the non-shielding cohort may have strictly self-isolated, we are following principles of analysis by Intention to Treat or Treatment Allocated [47] as this is most suited to a pragmatic evaluation context where study findings relate to how the intervention was implemented, not just how it was intended.

Public involvement

People affected by the shielding policy have been directly involved throughout study development. Two were co-applicants on the funding proposal and are members of the Research Management Group overseeing study implementation (LG, LD). Academic co-applicants (HS and BAE) were personally directly or indirectly affected by implementation of the shielding policy. We will recruit a Patient Advisory Panel of up to eight individuals affected by the shielding policy to supplement public input and support LD and LG. Public contributors will be involved at all stages of study delivery and dissemination. We will recruit two additional individuals to join the independent Study Steering Committee of clinical, policy, academic, methodological and public contributor experts. We will provide honoraria, briefings and other support as needed in line with best practice and report public involvement in our outputs [48-50]. We have a named lead for public involvement in the team (BAE) who brings expertise and experience to this role.

Study management and delivery

We will implement a comprehensive strategic and operational management, delivery and oversight infrastructure: Research Management Group (RMG) (research staff, all co-applicants), bi-monthly; Patient Advisory Panel (eight public contributors; chaired by a public contributor from, and reporting to, the RMG), quarterly; independent Study Steering Committee (clinical, policy, academic, methodological and public contributor experts), half-yearly; Core Research Group, reporting to RMG, two - four weeks .

ETHICS AND DISSEMINATION

We have ethical permission from the Newcastle North Tyneside 2 Research Ethics Committee (IRAS 295050) and approval under the SAIL independent Information Governance Review Panel (IGRP) project number 0911.

For peer review only - http://bmjopen.bmj.com/site/about/guidelines.xhtml

We will prepare a publication and engagement plan, informed by the insight and expertise of our clinical, academic, public and policy co-applicants to reach a range of audiences.

We will disseminate results directly to policy makers through the Welsh Government COVID-19 Technical Advisory Group (TAG), and the UK government Scientific Advisory Group for Emergencies (SAGE) and its related subgroups.

We will publish in peer-reviewed scientific journals and present at scientific and policy conferences (for a recent example, see https://hsruk.org/conference/conference-2021/workshops/pros-and-cons-shielding-vulnerable-people-public-health-policy given to the 2021 Health Services Research UK Conference).

Our public contributors will lead production of accessible summaries of findings which we will publish online (http://www.primecentre.wales/), share with our strong public and patient networks and promote through our social media networks.

In this first national evaluation of the effects of the UK COVID-19 shielding policy, we will contribute evidence for the role of immunity in prediction of outcome. Alongside emerging evidence from other studies undertaken through the National Core Studies Immunity programme, the proposed research will support the UK in preparation for future pandemics particularly concerning the health and safety of the most vulnerable members of society.

REFERENCES

1. Dowd JB, Andriano L, Brazel DM, Rotondi V, Block P, Ding XJ, Liu Y, Mills MC. Demographic science aids in understanding the spread and fatality rates of COVID-19. *Proceedings of the National Academy of Sciences.* 2020*;* 117(18): 9696-9698
2. Deng G, Yin M, Chen X., Zeng, F. Clinical determinants for fatality of 44,672 patients with COVID-19. *Crit. Care*. 2020. 24
3. Docherty AB et al. Features of 16,749 hospitalised UK patients with COVID-19 using the ISARIC WHO Clinical Characterisation Protocol. *BMJ.* 2020; 369:m1985 | doi: 10.1136/bmj.m1985
4. Williamson EJ, Walker AJ, Bhaskaran K.*et al.* OpenSAFELY: factors associated with COVID-19 death in 17 million patients. *Nature*. 2020.
5. Smith G.D., Spiegelhalter D. Shielding from COVID-19 should be stratified by risk. BMJ.2020;369:m2063

For peer review only - http://bmjopen.bmj.com/site/about/guidelines.xhtml

6. Public Health England. Guidance on protecting people who are clinically extremely vulnerable from COVID-19. Available from: https://www.gov.uk/government/publications/guidance-on-shielding-and-protecting-extremely-vulnerable-persons-from-covid-19/guidance-on-shielding-and-protecting-extremely-vulnerable-persons-from-covid-19. Accessed: 13 July 2020
7. Welsh Government. Guidance on protecting people defined on medical grounds as clinically extremely vulnerable from coronavirus (COVID-19) – previously known as 'shielding'. Available from: https://gov.wales/guidance-protecting-people-defined-medical-grounds-clinically-extremely-vulnerable-coronavirus. Accessed: 13 July 2020
8. Controlling COVID-19 through enhanced population surveillance and intervention (ConCOV: Swansea University. Available from: https://popdatasci.swan.ac.uk/swansea-university-led-team-secures-funding-from-covid-19-rapid-response-call/. Accessed: 13 July 2020
9. Written Statement: Coronavirus (COVID-19) – Shielding Update (5 May 2020) | GOV.WALES. Accessed 2 November 2021
10. https://www.ons.gov.uk/peoplepopulationandcommunity/healthandsocialcare/conditionsanddiseases/bulletins/coronavirusandshieldingofclinicallyextremelyvulnerablepeopleinengland/9julyto16july2020 Accessed 2 November 2021
11. https://gov.wales/sites/default/files/statistics-and-research/2020-07/shielded-patient-list-in-wales-during-the-coronavirus-covid-19-pandemic-as-at-6-july-2020-830.ods Accessed 2 November 2021
12. National Audit Office. Protecting and supporting the clinically extremely vulnerable during lockdown. Report by the Comptroller and Auditor General 2021. London: Ministry of Housing, Communities & Local Government, Department of Health & Social Care
13. European Centre for Disease Prevention and Control (ECDC). Guidelines for the use of non-pharmaceutical measures to delay and mitigate the impact of 2019-nCoV. Stockholm: ECDC; 2020. Available from: https://www.ecdc.europa.eu/en/publications-data/guidelines-use-non-pharmaceutical-measures-delay-and-mitigate-impact-2019-ncov Accessed: 13 July 2020
14. Information from the European Centre for Disease Prevention and Control (ECDC). Coronavirus disease 2019 (COVID-19) in the EU/EEA and the UK – eighth update 8 April 2020. Available from: https://www.ecdc.europa.eu/sites/default/files/documents/covid-19-rapid-risk-assessment-coronavirus-disease-2019-eighth-update-8-april-2020.pdf Accessed: 13 July 2020

For peer review only - http://bmjopen.bmj.com/site/about/guidelines.xhtml

15. Evans BA, Akbari A, Bethell L et al. Response to 'Covid-19: Government's shielding scheme failed thousands of clinically extremely vulnerable people, say MPs' *BMJ* 2021; 373 doi: https://doi.org/10.1136/bmj.n1033 (Published 20 April 2021)
16. Eregie C.O. COVID-19 Pandemic and stratified shielding: Some more matters for the works *BMJ* 2020;369:m2063 doi: 10.1136/bmj.m2063 (Published 28 May 2020)
17. Hume E, Armstrong M, Manifield J, *et al*. Impact of COVID-19 shielding on physical activity and quality of life in patients with COPD. *Breathe* 2020; 16: 200231.
18. Bevan Foundation (2020) Lessons from lockdown: the experience of shielding. Available at: https://www.bevanfoundation.org/resources/experience-of-shielding/. Accessed: 07 September 2021
19. Bevan Foundation (2021) Social isolation and loneliness: the impact of shielding in Merthyr Tydfil. Available at: https://www.bevanfoundation.org/resources/social-isolation-loneliness-shielding-in-merthyr-tydfil/ Accessed: 07 September 2021
20. Kemp O., Home G.A., Soutar R. The psychological impact of COVID19 on a shielding high-risk cohort. Scottish Medical Journal 2020. Vol 65(4) 120-122
21. Davies AR, Song J, Bentley L, Akbari A, Smith T, Carter B, John G, Trigg L, Parry G, Dundon J, Cross L. (2021). *COVID-19 in Wales: The impact on levels of health care use and mental health of the clinically extremely vulnerable.* Cardiff: Public Health Wales.
22. Jani B.D., Ho F.K., Lowe D.J., Traynor J.P., MacBride-Steward S.P., Mark P.B., Mair F.S., Pell J.P. Comparison of COVID-19 outcomes among shielded and non-shielded populations. Scientific Reports (2021) 11:15278. https://doi.org/10.1038/s41598-021-94630-6
23. McKeigue PM, McAllister DA, Caldwell D, Gribben C, Bishop J, McGurnaghan S, Armstrong M, Delvaux J, Colville S, Hutchinson S, Robertson C, Lone N, McMenamin J, Goldberg D, Colhoun HM. Relation of severe COVID-19 in Scotland to transmission-related factors and risk conditions eligible for shielding support: REACT-SCOT case-control study. BMC Med. 2021 Jun 23;19(1):149. doi: 10.1186/s12916-021-02021-5. PMID: 34158021; PMCID: PMC8219469.Oliver, K., Lorenc, T., Tinkler, J. et al. Understanding the unintended consequences of public health policies: the views of policymakers and evaluators. BMC Public Health 19, 1057 (2019). https://doi.org/10.1186/s12889-019-7389-6

For peer review only - http://bmjopen.bmj.com/site/about/guidelines.xhtml

24. Oliver, K., Lorenc, T., Tinkler, J. et al. Understanding the unintended consequences of public health policies: the views of policymakers and evaluators. BMC Public Health 19, 1057 (2019). https://doi.org/10.1186/s12889-019-7389-6

25. Snooks H, Bailey-Jones K, Burge-Jones D, et al Effects and costs of implementing predictive risk stratification in primary care BMJ Quality & Safety 2019;28:697-705.

26. Lyons J, Akbari A, Torabi F, Davies G, North L, Griffiths R, Bailey R, Hollinghurst J, Fry R, Turner S, Thompson D, Rafferty J, Mizen A, Orton C, Ellwood-Thompson S, Au-Yeung L, Cross L, Gravenor M, Brophy S, Lucini B, John A, Szakmany T, Davies J, Davies C, Williams C, Emmerson C, Cottrell S, Connor T, Taylor C, Pugh R, Diggle PJ, John G, Scourfield S, Hunt J, Cunningham AM, Helliwell K, Lyons RA. Understanding and responding to COVID19 in Wales: protocol for a privacy protecting data platform for enhanced epidemiology and evaluation of interventions. BMJ Open 2020;10:e043010. doi:10.1136/ bmjopen-2020-043010

27. Welsh Government (2020) Further action to protect people at highest risk from coronavirus | GOV.WALES. Accessed 29 November 2021

28. Welsh Government (2020) Written Statement: Coronavirus (COVID-19) – Shielding Update – changes to advice (1 June 2020) | GOV.WALES Accessed 29 November 2021

29. Welsh Government (2020) Written statement: An update on shielding and adopting the guidance of the Royal College of Paediatrics and Child Health (RCPCH) (16 July 2020) | GOV.WALES Accessed 29 November 2021

30. Welsh Government (2021) Written Statement: Advice to those who are Clinically Extremely Vulnerable – extension to period advice covers (29 January 2021) | GOV.WALES Accessed 29 November 2021

31. Welsh Government (2021) Guidance on protecting people defined on medical grounds as clinically extremely vulnerable from coronavirus (COVID-19) – previously known as 'shielding' [HTML] | GOV.WALES Accessed 29 November 2021

32. Mills, T., Lawton, R. & Sheard, L. Advancing complexity science in healthcare research: the logic of logic models. *BMC Med Res Methodol. 2019;* **19,** 55

33. Cochrane Infectious Diseases Group. Developing Logic Models. 2016; Effective Healthcare Consortium

34. Ware J Jr, Kosinski M, Keller SD. A 12-Item Short-Form Health Survey: construction of scales and preliminary tests of reliability and validity. *Med Care*. 1996;34(3):220-233.

For peer review only - http://bmjopen.bmj.com/site/about/guidelines.xhtml

35. Spitzer RL, Kroenke K, Williams JB. Validation and Utility of a Self-Report Version of PRIME-MD: The PHQ Primary Care Study. Primary Care Evaluation of Mental Disorders. Patient Health Questionnaire. *JAMA.* 1999 Nov 10;282(18):1737-44

36. Spitzer RL, Kroenke K, Williams JB. Loewe B A Brief Measure for Assessing Generalized Anxiety Disorder The GAD-7 *Arch Intern Med.* 2006;166(10):1092-1097.

37. Cooper J, Williams H, Hibbert P, Edwards A, Butt A, Wood F, Parry G, Smith P, Sheikh A, Donaldson L, Carson-Stevens A. Classification of patient-safety incidents in primary care. Bull World Health Organ. 2018 Jul 1;96(7):498-505.

38. Wright J, Lawton R, O'Hara J, et al. Improving patient safety through the involvement of patients: development and evaluation of novel interventions to engage patients in preventing patient safety incidents and protecting them against unintended harm. Southampton (UK): NIHR Journals Library 2016.

39. Sampson H, Johannessen IA. Turning on the tap: the benefits of using 'real-life' vignettes in qualitative research interviews. *Qualitative Research*. 2020;20(1):56-72. doi:10.1177/1468794118816618

40. Gale, N.K., Heath, G., Cameron, E. *et al.* Using the framework method for the analysis of qualitative data in multi-disciplinary health research. *BMC Med Res Methodol* **13,** 117 (2013). https://doi.org/10.1186/1471-2288-13-117

41. Srivastava, A. & Thomson, S. B. (2009). Framework Analysis: A Qualitative Methodology for Applied Research Note Policy Research. JOAAG, Vol. 4. No. 2

42. Swansea Trials Unit (2020). STU-SOP-DMS-004 – Standard Operating Procedure on Creating a Statistical Analysis Plan, Swansea University.

43. Scurr, Martin J., Wioleta M. Zelek, George Lippiatt, Michelle Somerville, Stephanie E. A. Burnell, Lorenzo Capitani, Kate Davies, et al. 2021. 'Whole Blood-Based Measurement of SARS-CoV-2-Specific T Cell Responses Reveals Asymptomatic Infection and Vaccine Efficacy in Healthy Subjects and Patients with Solid Organ Cancers'. *MedRxiv*, January, 2021.06.02.21258218. https://doi.org/10.1101/2021.06.02.21258218

44. Braun, V., & Clarke, V. (2012). Thematic analysis. In H. Cooper, P. M. Camic, D. L. Long, A. T. Panter, D. Rindskopf, & K. J. Sher (Eds.), *APA handbook of research methods in psychology, Vol. 2. Research designs: Quantitative, qualitative, neuropsychological, and biological* (pp. 57–71). American Psychological Association. https://doi.org/10.1037/13620-004]

For peer review only - http://bmjopen.bmj.com/site/about/guidelines.xhtml

45. Personal Social Services Research Unit (PSSRU). Unit Costs of Health and Social Care 2020. 2020 [Available from: www.pssru.ac.uk/project-pages/unit-costs/unit-costs-2020.
46. National Health Service (NHS). NHS Reference costs 2019 to 2020. 2021. (Updated: June 2021) Available at: https://www.england.nhs.uk/national-cost-collection/. Accessed: 21 July 2021.
47. Gupta, S.K., 2011. Intention-to-treat concept: a review. *Perspectives in clinical research*, *2*(3), p.109.
48. Evans BA, Gallanders J, Griffiths L, Harris-Mayes R, James M, Jones S, Joseph-Williams N, Nettle M, Rolph M, Snooks H, Wallace C, Edwards A and on behalf of the SUPER group and PRIME Centre Wales Public involvement and engagement in primary and emergency care research: the story from PRIME Centre Wales IJPDS 2020 https://doi.org/10.23889/ijpds.v5i3.1363
49. UK Standards for Public Involvement. Available at https://sites.google.com/nihr.ac.uk/pi-standards/home. Accessed 2 November 2021
50. Staniszewska, S., Brett, J., Simera, I., et al. GRIPP2 reporting checklists: tools to improve reporting of patient and public involvement in research
BMJ 2017; 358 doi: https://doi.org/10.1136/bmj.j3453

Authors' contributions:

BAE drafted the manuscript with editorial input from all authors – AA, RB, LB, SB, AC-S, LD, AE, AJ, SJ, MK, JL, RAL, AP, BS, CAT, AW, TW, HS. The research idea was conceived by HS and developed by all authors. All authors read and approved the final manuscript.

For peer review only - http://bmjopen.bmj.com/site/about/guidelines.xhtml

Acknowledgments:

This study makes use of anonymised data held in the Secure Anonymised Information Linkage (SAIL) Databank. This work uses data provided by patients and collected by the NHS as part of their care and support. We would also like to acknowledge all data providers who make anonymised data available for research. We wish to acknowledge the collaborative partnership that enabled acquisition and access to the de-identified data, which led to this output. The collaboration was led by the Swansea University Health Data Research UK team under the direction of the Welsh Government Technical Advisory Cell (TAC) and includes the following groups and organisations: the SAIL Databank, Administrative Data Research (ADR) Wales, Digital Health and Care Wales (DHCW), Public Health Wales, NHS Shared Services Partnership (NWSSP) and the Welsh Ambulance Service Trust (WAST). All research conducted has been completed under the permission and approval of the SAIL independent Information Governance Review Panel (IGRP) project number 0911.

Funding statement:

This work is supported by the National Core Studies Immunity (NCSi4P) Programme (award number UoB WT Ref: 1745068). This work was supported by the Con-COV team funded by the Medical Research Council (grant number: MR/V028367/1). This work was supported by Health Data Research UK, which receives its funding from HDR UK Ltd (HDR-9006) funded by the UK Medical Research Council, Engineering and Physical Sciences Research Council, Economic and Social Research Council, Department of Health and Social Care (England), Chief Scientist Office of the Scottish Government Health and Social Care Directorates, Health and Social Care Research and Development Division (Welsh Government), Public Health Agency (Northern Ireland), British Heart Foundation (BHF) and the Wellcome Trust. This work was supported by the ADR Wales programme of work (award number ES/S007393/1). The ADR Wales programme of work is aligned to the priority themes as identified in the Welsh Government's national strategy: Prosperity for All. ADR Wales brings together data science experts at Swansea University Medical School, staff from the Wales Institute of Social and Economic Research, Data and Methods (WISERD) at Cardiff University and specialist teams within the Welsh Government to develop new evidence which supports Prosperity for All by using the SAIL Databank at Swansea University, to link and analyse anonymised data. ADR Wales is part of the Economic and Social Research Council (part of UK Research and Innovation) funded ADR UK (grant ES/S007393/1).

For peer review only - http://bmjopen.bmj.com/site/about/guidelines.xhtml

This work was supported by the Wales COVID-19 Evidence Centre, funded by Health and Care Research Wales.

Availability of data and materials:

The data used in this study are available in the SAIL Databank at Swansea University, Swansea, UK, but as restrictions apply they are not publicly available. All proposals to use SAIL data are subject to review by an independent Information Governance Review Panel (IGRP). Before any data can be accessed, approval must be given by the IGRP. The IGRP gives careful consideration to each project to ensure proper and appropriate use of SAIL data. When access has been granted, it is gained through a privacy protecting safe haven and remote access system referred to as the SAIL Gateway. SAIL has established an application process to be followed by anyone who would like to access data via SAIL at https://www.saildatabank.com/application-process

Competing interests:

Ronan Lyons, Stephen Jolles, Ann John and Adrian Edwards are members of the Welsh Government COVID-19 Technical Advisory Group. Ann John is also co-Chair of the Scientific Pandemic Insights Group on Behaviours (SPI-B) which is a subgroup of the Scientific Advisory Group for Emergencies (SAGE) advising the UK government. Stephen Jolles is also a member of the Welsh Government Testing TAG and Cardiff University COVID Strategic Advisory Board (SAB). No other authors have competing interests.

Exclusive licence

I, the Submitting Author has the right to grant and does grant on behalf of all authors of the Work (as defined in the below author licence), an exclusive licence and/or a non-exclusive licence for contributions from authors who are: i) UK Crown employees; ii) where BMJ has agreed a CC-BY licence shall apply, and/or iii) in accordance with the terms applicable for US Federal Government officers or employees acting as part of their official duties; on a worldwide, perpetual, irrevocable, royalty-free basis to BMJ Publishing Group Ltd ("BMJ") its licensees and where the relevant Journal is co-owned by BMJ to the co-owners of the Journal, to publish the Work in BMJ Open and any other BMJ products and to exploit all rights, as set out in our licence.

The Submitting Author accepts and understands that any supply made under these terms is made by BMJ to the Submitting Author unless you are acting as an employee on behalf of your employer or a postgraduate student of an affiliated institution which is paying any applicable article publishing charge ("APC") for Open Access articles. Where the Submitting Author wishes to make the Work available on an Open Access basis (and intends to pay the relevant APC), the terms of reuse of such Open Access shall be governed by a Creative Commons licence – details of these licences and which Creative Commons licence will apply to this Work are set out in our licence referred to above.

For peer review only

Tables and figures

For peer review only

For peer review only - http://bmjopen.bmj.com/site/about/guidelines.xhtml

Table 1: Summary of methods and outputs against study objectives

**Objective**	**Method**	**Output**
1. Capture rationale for UK shielding.	Interviews with policy makers.	Logic model to describe components, outcomes and mechanisms of shielding.
2. Assess effects of shielding in the general population and subgroups in terms of deaths, hospitalisations, safety and self-reported health.	Comparison of anonymised routine data between shielded population and comparator group.	Comparative outcomes for shielded and matched non-shielded people:<br>COVID-19 tests, PCR-confirmed infections and deaths.<br>All-cause mortality.<br>Emergency Department attendances, emergency hospital admissions, days spent in hospital, ICU admissions and days spent in ICU.
	Questionnaire to stratified random sample of intervention and control groups.	Self-reported health related quality-of-life, anxiety, depression and loneliness.
3. Assess the infection and immunity within the shielded and control populations as a whole.	Analysis of routine records of blood tests in shielded population.<br><br>Analysis of blood samples from stratified random subsample of intervention and control groups.	Detailed analysis of COVID-19 antigen and antibody test results within the shielded and comparator populations, to understand:<br>• effects of shielding on infection and immunity across the population and for clinical subgroups including cancer.<br>• impact of immunological status on outcomes in the shielded and non-shielded population.
4. Explore behaviour, adherence, and safety concerns relating to shielding.	Interviews with 40 individuals and carers/household members who were shielded.	Experiences of shielded people and their carers or household members during the COVID-19 lockdown, including behaviour, emotional effects and safety concerns.
5. Assess costs of the shielding intervention against its consequences.	Investigation of costs and cost-consequences of managing and delivering shielding.	Costs of intervention implementation and subsequent healthcare resource use compared to consequences and outcomes, net monetary benefit.
6. Understand the experiences and views of health care providers in relation to the shielding intervention and perceived effects, including healthcare associated harms.	Interviews with 30 community-based health professionals.	Experiences of clinicians delivering care to shielded population during pandemic.

For peer review only - http://bmjopen.bmj.com/site/about/guidelines.xhtml

Table 2: Shielding advice given to clinically extremely vulnerable (CEV) people

**Key points**	Do not leave the house to go to work or to see other people
	Avoid being in the same room as another person
	Keep three steps away from another person in the home
	Avoid sharing kitchen, bathroom or bedroom facilities with others in the home
	Eat meals separately from other household members
	Be aware that hospital appointments and treatment may be postponed or cancelled
**Support**	Letters included details of available support, including how to obtain food, prescriptions and other information
**Repeat advice**	Letters were sent to clinically extremely vulnerable people throughout the pandemic which updated current advice.<br>Correspondence after the first 12 week shielding period reflected an easing of some of the above points until January 2021 when shielding advice was reinstated. Advice remained, with some amendments, until August 2021. Shielding officially ended in March 2022.

For peer review only - http://bmjopen.bmj.com/site/about/guidelines.xhtml

Figure 1: Data Flow Visualisation

Figure 2: Study Participant Recruitment Flow Chart

For peer review only

For peer review only - http://bmjopen.bmj.com/site/about/guidelines.xhtml

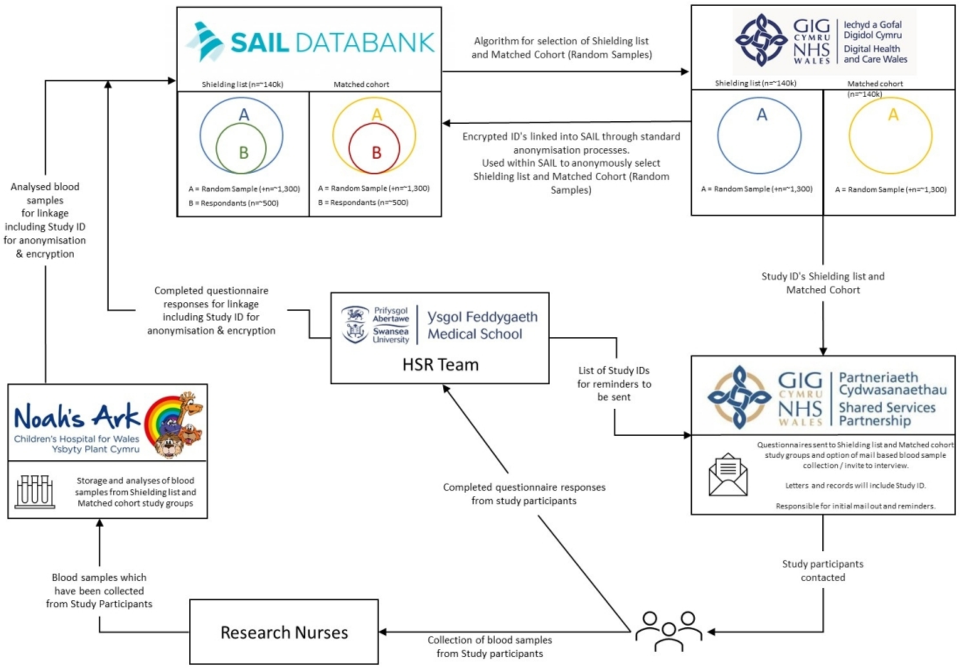

Figure 1: Data Flow Visualisation

131x90mm (300 x 300 DPI)

EVITE study participant recruitment flowchart

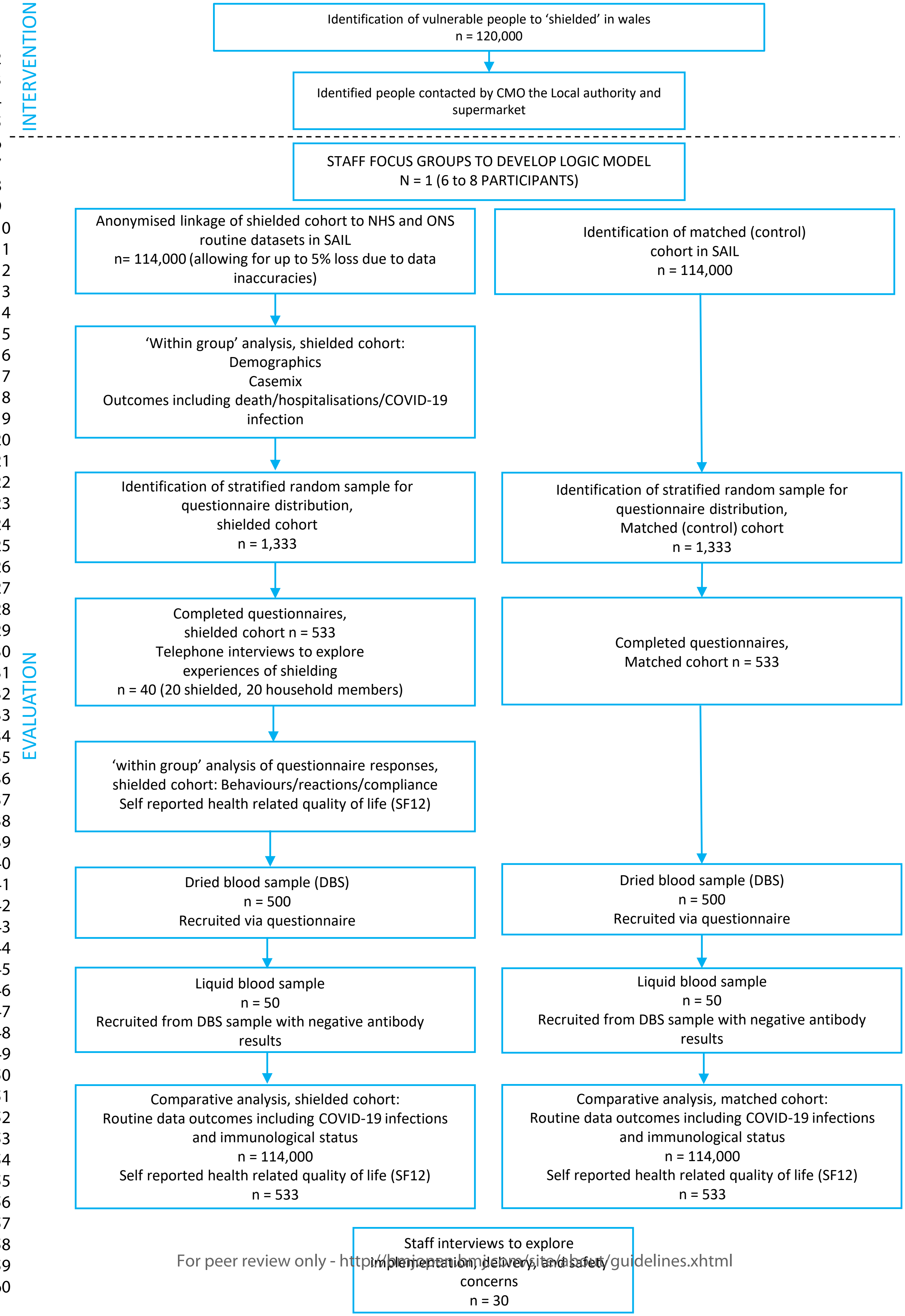

Appendix 1

EVITE Immunity

Prifysgol Abertawe Swansea University | Ysgol Feddygaeth Medical School

GIG CYMRU NHS WALES | Iechyd a Gofal Digidol Cymru Digital Health and Care Wales

Address line 1
Address line 2
Address line 3
Address line 4
Address line 5

Dear [example – Mr David Jones –]

Please cut or tear to remove the **top** section of **this page** before returning

✂ -

(your study ID is 12345)

We would like to invite you to help us with our research study looking at the effects of public health policy in Wales during the COVID-19 pandemic.

We are asking you to help by completing the questionnaire and sending it back to us using the enclosed prepaid envelope. We would like to know about how you have been feeling over the last 12 months. The questionnaire should take around 20 minutes to complete. If you are happy to take part, please complete the questionnaire and return to the researchers using the supplied prepaid envelope. You can use the QR code to complete it online or by going to the website address below. We are not able to provide the questionnaire in Welsh as some parts of it have only been tested and validated for research in English.

Your input in this study is really valuable and we appreciate your time in completing the questionnaire.

Kind regards,

Professor Helen Snooks, Chief Investigator, Dr Victoria Williams, Study Manager, and the EVITE Immunity Study Team

If you would prefer to complete the questionnaire online, please scan in the QR code or visit the following website:
https://swansea.onlinesurveys.ac.uk/evite-immunity-questionnaire-su

QR CODE TO ONLINE SURVEY

Please contact us for further information or assistance to complete the questionnaire
Phone: 01792 513279
Email: EVITEIMMUNITY2@Swansea.ac.uk

IRAS 295050

For peer review only - http://bmjopen.bmj.com/site/about/guidelines.xhtml

For peer review only

Annwyl Sir/madam

Hoffem eich gwahodd i'n helpu ni gyda'n hastudiaeth ymchwil sy'n ystyried effeithiau polisi iechyd cyhoeddus yng Nghymru yn ystod pandemig Covid-19.

Gofynnwn i chi ein helpu ni trwy gwblhau'r holiadur a'i ddychwelyd atom ni gan ddefnyddio'r amlen wedi'i rhagdalu amgaeedig. Hoffem wybod sut rydych chi wedi bod yn teimlo dros y 12 mis diwethaf. Dylai gymryd tuag 20 munud i gwblhau'r holiadur. Os ydych chi'n hapus i gymryd rhan, llenwch yr holiadur a'i ddychwelyd at yr ymchwilwyr gan ddefnyddio'r amlen wedi'i rhagdalu amgaeedig. Gallwch chi ddefnyddio'r côd QR i'w lenwi ar-lein neu drwy fynd i gyfeiriad y wefan isod. Ni allwn ddarparu'r holiadur yn Gymraeg am fod rhai rhannau wedi'u profi a'u dilysu ar gyfer ymchwil yn Saesneg yn unig.

Mae eich mewnbwn i'r astudiaeth hon yn werthfawr iawn ac rydym yn gwerthfawrogi'ch amser yn llenwi'r holiadur.

For peer review only

Cofion cynnes,

Yr Athro Helen Snooks, Prif Ymchwilydd, Dr Victoria Williams, Rheolwr yr Astudiaeth, a Thîm Astudiaeth Imiwnedd EVITE

Os byddai'n well gennych chi lenwi'r holiadur ar-lein, sganiwch y côd QR neu ewch i'r wefan ganlynol: https://swansea.onlinesurveys.ac.uk/evite-immunity-questionnaire-su

CÔD QR AR GYFER YR AROLWG AR-LEIN

Cysylltwch â ni am gymorth neu wybodaeth pellach i'w gwbwlhau yr ymholiadur os gwelwch yn dda.

Ffôn: 01792 513279

E-bost: EVITEIMMUNITY2@Swansea.ac.uk

For peer review only - http://bmjopen.bmj.com/site/about/guidelines.xhtml

Study ID Number: | | | | | |

[Please insert your Study ID as shown on the first page of the cover letter]

Section 1

1. Did you at any time receive a shielding letter or text message from the NHS or Chief Medical Officer saying that you have been identified as someone at risk of severe illness (COVID-19) if you catch Coronavirus, because you have an underlying disease or health condition? Please select a box that applies.

Yes	
No	
Don't know	
Don't want to answer	

2. **After the first lockdown was announced in March 2020,** on average can you recall how many days you met up with any of your family or friends who do not live with you?

Everyday	
4-6 days each week	
2-3 days each week	
1 day each week	
Less than 1 day per week	
Don't know	
Don't want to answer	

3. **Over the last 2 weeks,** on average can you recall how many days you met up with any of your family or friends who do not live with you?

Everyday	
4-6 days each week	
2-3 days each week	
1 day each week	
Less than 1 day per week	
Don't know	
Don't want to answer	

For peer review only

For peer review only - http://bmjopen.bmj.com/site/about/guidelines.xhtml

4. The following questions are about what you did at the time of the initial announcement of lockdown in March and during the last 2 weeks. Please select one response for each question and each time point (i.e. March and the last 2 weeks)

On a scale of 1 to 5, where 1 is Never and 5 is Always, please let us know how often you did the below.

	**During the initial announcement of lockdown in March 2020**	**During the last 2 weeks**
Strictly avoided contact with someone who displayed symptoms of coronavirus	1. Never<br>2. Rarely<br>3. Sometimes<br>4. Very often<br>5. Always	1. Never<br>2. Rarely<br>3. Sometimes<br>4. Very often<br>5. Always
Stayed at home	1. Never<br>2. Rarely<br>3. Sometimes<br>4. Very often<br>5. Always	1. Never<br>2. Rarely<br>3. Sometimes<br>4. Very often<br>5. Always
Felt scared to go outside	1. Never<br>2. Rarely<br>3. Sometimes<br>4. Very often<br>5. Always	1. Never<br>2. Rarely<br>3. Sometimes<br>4. Very often<br>5. Always
Attended any gathering (Including gatherings of friends and families in private spaces e.g. family homes, weddings and religious services)	1. Never<br>2. Rarely<br>3. Sometimes<br>4. Very often<br>5. Always	1. Never<br>2. Rarely<br>3. Sometimes<br>4. Very often<br>5. Always
Went out for shopping, leisure or travel	1. Never<br>2. Rarely<br>3. Sometimes<br>4. Very often<br>5. Always	1. Never<br>2. Rarely<br>3. Sometimes<br>4. Very often<br>5. Always
Kept in touch using remote technology e.g. phone, internet and social media	1. Never<br>2. Rarely<br>3. Sometimes<br>4. Very often<br>5. Always	1. Never<br>2. Rarely<br>3. Sometimes<br>4. Very often<br>5. Always
Used telephone or online services to contact your GP or other essential services	1. Never<br>2. Rarely<br>3. Sometimes<br>4. Very often<br>5. Always	1. Never<br>2. Rarely<br>3. Sometimes<br>4. Very often<br>5. Always
Regularly washed your hands with soap and water for 20 seconds	1. Never<br>2. Rarely<br>3. Sometimes<br>4. Very often<br>5. Always	1. Never<br>2. Rarely<br>3. Sometimes<br>4. Very often<br>5. Always

For peer review only

5. The following questions are about what you did after the initial announcement of lockdown in March and during the last 2 weeks. Please select one response for each question and each time point (i.e. March and the last 2 weeks)

On a scale of 1 to 5 , <u>where 1 is Never and 5 is Always</u>, please let us know how often you did the below within your household (including visitors and carers)

	**During the initial announcement of lockdown in March 2020**	**During the last 2 weeks**
Minimised the time you spent with others from your household in shared spaces (kitchen, bathroom and sitting areas)	1. Never<br>2. Rarely<br>3. Sometimes<br>4. Very often<br>5. Always	1. Never<br>2. Rarely<br>3. Sometimes<br>4. Very often<br>5. Always
Kept shared spaces well ventilated	1. Never<br>2. Rarely<br>3. Sometimes<br>4. Very often<br>5. Always	1. Never<br>2. Rarely<br>3. Sometimes<br>4. Very often<br>5. Always
Used separate towels from the rest of your household	1. Never<br>2. Rarely<br>3. Sometimes<br>4. Very often<br>5. Always	1. Never<br>2. Rarely<br>3. Sometimes<br>4. Very often<br>5. Always
Used a separate bathroom from the rest of your household or cleaned the bathroom after every use	1. Never<br>2. Rarely<br>3. Sometimes<br>4. Very often<br>5. Always	1. Never<br>2. Rarely<br>3. Sometimes<br>4. Very often<br>5. Always
Avoided using the kitchen when others are present and ensured kitchenware is cleaned thoroughly	1. Never<br>2. Rarely<br>3. Sometimes<br>4. Very often<br>5. Always	1. Never<br>2. Rarely<br>3. Sometimes<br>4. Very often<br>5. Always

For peer review only

The following questions are to help us understand any safety concerns you have had over the last 12 months.

6. **While trying to access or receive NHS care during the coronavirus pandemic have you experienced something that you thought was a 'safety concern'?**

A safety concern can be any event or situation where you think something went wrong or not as good as expected whilst receiving or trying to receive health care. The event or situation might have led to harm or you feel could result in harm in the future if the concern is not addressed. By harm, we mean the event or situation is impacting you or another person physically (like a worsening of symptoms or your condition, or any disabling complications), emotionally (upset or loss of trust) or psychologically (any worsening of your mental health).

Yes	
No	
Don't know	
Don't want to answer	

IF YES GO TO **QUESTION 7**, IF NO GO TO **SECTION 2.**

7. **What did the safety concerns relate to and on a scale from 1 (not serious at all) to 10 (extremely serious), how serious do you think your safety concern was? Please rate any concern that you experienced by selecting the appropriate number on the scale.**

Diagnosis of your problem	1 2 3 4 5 6 7 8 9 10
Access to the NHS service you needed	1 2 3 4 5 6 7 8 9 10
Tests or procedures that were performed (e.g. blood tests, scans)	1 2 3 4 5 6 7 8 9 10
Medication, vaccines or treatment	1 2 3 4 5 6 7 8 9 10
Delay or cancellation of treatment for pre-existing condition	1 2 3 4 5 6 7 8 9 10
Communication between you and the healthcare professional(s)	1 2 3 4 5 6 7 8 9 10
Communication and co-ordination between different healthcare professionals	1 2 3 4 5 6 7 8 9 10
Concerns specific to the coronavirus outbreak (e.g. personal protective equipment)	1 2 3 4 5 6 7 8 9 10
Information provided	1 2 3 4 5 6 7 8 9 10
Vaccination	1 2 3 4 5 6 7 8 9 10
Other	1 2 3 4 5 6 7 8 9 10
If other, please specify ______________________________.	1 2 3 4 5 6 7 8 9 10

For peer review only - http://bmjopen.bmj.com/site/about/guidelines.xhtml

8. **In which health care setting(s) did the safety concern(s) take place? Please tick all that apply**.

GP services (e.g. GP, nurse appointment, health visitor)	
A&E	
Routine outpatient services (e.g. appointment with hospital doctor/nurse specialist, consultant, physiotherapy, speech therapy, dialysis, mental health services)	
Inpatient services (e.g. routine surgery, admission to hospital)	
Midwifery and maternity services	
District nurse	
Optician	
Pharmacist	
Dentist	
NHS 111 service	
COVID-19 Vaccination services	
Other	
If other, please specify	

9. **Is there anything else that you would like to tell us about your safety concern(s)?**

Yes	
No	
If yes, please specify	________

If you would like advice on reporting a patient safety concern, please visit:
Wales: https://www.avma.org.uk/wp-content/uploads/Complaints-Wales.pdf

For peer review only

Section 2

Over the last two weeks, how often have you been bothered by any of the following problems?

	Not at all	Several Days	More than half the days	Nearly everyday
Little interest of pleasure in doing things				
Feeling down, depressed, or hopeless				
Trouble falling or staying asleep, or sleeping too much				
Feeling tired or having little energy				
Poor appetite or overeating				
Feeling bad about yourself-or that you are a failure or have let yourself or your family down				
Trouble concentrating on things, such as reading the newspaper or watching television				
Moving or speaking so slowly that other people could have noticed? Or the opposite- being so fidgety or restless that you have been moving around a lot more than usual				
Thoughts that you would be better off dead, or of hurting yourself in some way				

Section 3

Over the last 2 weeks, how often have you been bothered by any of the following problems?

	Not at all	Several Days	More than half the days	Nearly everyday
Feeling nervous, anxious or on edge				
Not being able to stop or control worrying				
Worrying too much about different things				
Trouble relaxing				
Being so restless that it is hard to sit still				
Becoming easily annoyed or irritable				
Feeling afraid as if something awful might happen				

For peer review only

Section 4

This section of the questionnaire asks for your views about your health. This information will help keep track of how you feel and how well you are able to do your usual activities. We will ask you how you have felt at different time points – two weeks ago and four weeks ago **Answer each question by choosing just one answer.** If you are unsure how to answer a question, please give the best answer you can.

1. **In general, would you say your health is:**

Excellent ☐
Very good ☐
Good ☐
Fair ☐
Poor ☐

The following questions are about activities you might do during a typical day. Does your health now limit you in these activities? If so, how much?

	YES, limited a lot	YES, limited a little	NO, not limited at all
2. **Moderate activities** such as digging in the garden, spring cleaning or other heavy housework, gentle swimming or cycling	☐	☐	☐
3. Climbing **several** flights of stairs	☐	☐	☐

During the past 4 weeks, have you had any of the following problems with your work or other regular daily activities as a result of your physical health?

	YES	NO
4. **Accomplished** less than you would like	☐	☐
5. Were **limited** in the kind of work or other activities	☐	☐

For peer review only

For peer review only - http://bmjopen.bmj.com/site/about/guidelines.xhtml

During the past 4 weeks have you had any of the following problems with your work or other regular daily activities as a result of any emotional problems (such as feeling depressed or anxious)?

	**YES**	**NO**
6. **Accomplished** less than you would like	☐	☐
7. Did work or activities **less carefully than usual**	☐	☐

8. During the past 4 weeks, how much did pain interfere with your normal work (including work outside the home and housework)?

Not at all ☐

A little bit ☐

Moderately ☐

Quite a bit ☐

Extremely ☐

These questions are about how you have been feeling during the past 4 weeks. For each question, please give the one answer that comes closest to the way you have been feeling.

How much of the time during the past 4 weeks…

	**All of the time**	**Most of the time**	**A good bit of the time**	**Some of the time**	**A little of the time**	**None of the time**
9. Have you felt calm and peaceful?	☐	☐	☐	☐	☐	☐
10. Did you have a lot of energy?	☐	☐	☐	☐	☐	☐
11. Have you felt down-hearted and blue?	☐	☐	☐	☐	☐	☐

For peer review only - http://bmjopen.bmj.com/site/about/guidelines.xhtml

12. **During the past 4 weeks, how much of the time has your physical health or emotional problems interfered with your social activities (like visiting friends, relatives, etc)?**

- All of the time ☐
- Most of the time ☐
- Some of the time ☐
- A little of the time ☐
- None of the time ☐

Section 5

Please indicate how often each of the statements below is descriptive of you:

	I often feel this way	I sometimes feel this way	I rarely feel this way	I never feel this way
I lack companionship	☐	☐	☐	☐
I feel part of a group of friends	☐	☐	☐	☐
I feel left out	☐	☐	☐	☐
I feel isolated	☐	☐	☐	☐
I am unhappy being so withdrawn	☐	☐	☐	☐
People are around me but not with me	☐	☐	☐	☐

For peer review only

Section 6

The following questions ask you about treatment for any infections you contracted during the past two years, before and during the pandemic.

1. Thinking back to March 2019 - March 2020, the year before the pandemic, how many times do you estimate you took a course of antibiotics?

	Please tick which applies
0	
1 – 3 times	
4 – 6 times	
7 – 10 times	
More than 10 times	
Part of daily/weekly medication regime	
Other (please give details)	

2. Thinking back to March 2020 – March 21, the year since the pandemic started, how many times do you estimate you took a course of antibiotics?

	Please tick which applies
0	
1 – 3 times	
4 – 6 times	
7 – 10 times	
More than 10 times	
Part of daily/weekly medication regime	
Other (please give details)	

For peer review only

Section 7

Please let us know if you have anything else you would like to say about your experience over the last 12 months

only

For peer review only - http://bmjopen.bmj.com/site/about/guidelines.xhtml

Have you had at least one COVID-19 vaccination?

Yes ☐

No ☐

Thank you for your time in completing this questionnaire. Your input is really valuable. Please could you provide us with some information on who completed the questionnaire:

1. I completed the questionnaire without assistance ☐
2. A carer helped me to complete the questionnaire ☐

Further Research

Your answers will be treated in the strictest confidence and will only be used for research purposes. Your responses may be used with other anonymised information about you. *If you do not want your data to be used in this way please tick this box:*

☐

Please let us know if you are happy for us to contact you regarding other aspects of this research. Please tick the below boxes if you give your consent for us to contact you regarding:

1. Taking blood samples ☐
2. Telephone interviews ☐

If you consented to either or both of the above please provide appropriate contact details:

Telephone number: ________________________

Email address: ______________________________________

For peer review only

Appendix 2

EVITE Immunity

Effects of shielding for Vulnerable people during COVID-19 pandemic on health and Immunity: a quasi-experimenTal Evaluation

Swansea University
Prifysgol Abertawe
Medical School
Ysgol Feddygaeth

Effects of shielding for Vulnerable people during COVID-19 pandemic on health outcomes, costs and Immunity including those with cancer: quasi-experimenTal Evaluation (EVITE Immunity)

Shielded population-interview questions- v1.0 10.05.21

Establish before the interview whether the person responding is someone who has been shielding, a carer, or both are being interviewed at once.

1. Can you tell me about your household please?
    - Live alone?
    - Live with others? How many/who?
    - Anyone else in your household also shielding?
        i. Were these other people also sent a shielding letter
        ii. were they family members who decided to shield with you
    - Does anyone regularly come to your home to provide care or support? If so, paid carer or informal/family carer?

2. Can you recall receiving the letter about shielding in March or April 2020?
    - Was it clear to you?
    - How did the letter make you feel?

3. Since April 2020, have you received any more information about shielding?
    - Letter/other contact
    - Any change in guidance?

4. Once you got the shielding letter, how did your life change?
    - Staying in?
    - Avoiding visitors?
    - Avoiding interaction with other household members?
    - What stayed the same for you after you got the shielding letter
    - What aspects of your life were better because you were shielding?

5. It has been more than a year since the shielding programme was introduced. Over that time, have you made any changes in how you have lived, eg starting to go out of the house more.
    - When did you change what you were doing? (clarify how long they felt they were shielding)
    - Why did you change?
    - Have things changed more than once – eg going out more in the summer, staying home more in the winter, influence of changing rules and lockdowns?
    - Do you behave differently now that shielding has ended,compared to before the pandemic?

For peer review only - http://bmjopen.bmj.com/site/about/guidelines.xhtml

EVITE Immunity

Effects of shielding for Vulnerable people during COVID-19 pandemic on health and Immunity: a quasi-experimenTal Evaluation

Swansea University
Prifysgol Abertawe
Medical School
Ysgol Feddygaeth

6. How have you felt about being part of the shielding programme?
    - Positive feelings – eg Safe, protected, doing your bit
    - Negative feelings – eg frustrated, confused, afraid, lonely, sad
    - Neutral - took no notice of it
    - Have your feelings changed over time?
    - Do you consider yourself to have 'been shielding yourself' or 'being shielded by others/community'?

7. If you live with other people, what impact has the shielding programme had on them?
    - Have they changed the way they behave?
    - Explore whether others in household shielded/did not shield; why did they make this decision; what difference did it make for you?

8. Did you get any additional support to help you with shielding?
    - eg food parcels, help from your employer

9. Have you had contact with the health service over the last year (GP/Hospital/NHS 111/ambulance service etc)?
    - To discuss Covid 19 risks/shielding
    - For other health reasons
    - Hesitant about contacting NHS/avoided contact
    - Any cancelled or delayed treatments or tests
    - How has your normal healthcare changed over the past year?

10. Do you know anyone who has had Covid 19?
    - Self
    - Someone they have had direct contact with in the last year
    - Someone they have not had contact with in the last year
    - If yes, did that affect how they felt about shielding?

For peer review only - http://bmjopen.bmj.com/site/about/guidelines.xhtml

Appendix 3

Effects of shielding for Vulnerable people during COVID-19 pandemic on health outcomes, costs and Immunity including those with cancer: quasi-experimenTal Evaluation (EVITE Immunity)

Healthcare Professionals
Interview questions - v1.0 10.05.21

1. What is your clinical role?
    - GP/hospital doctor – speciality/nurse/other

2. How were you first informed about the shielding programme for patients extremely vulnerable to Covid-19?
    - Letter/other contact
    - Was it clear?
    - Any change in guidance over time? Have you been kept informed of changes?

3. What has been your role in putting the shielding programme in place?
    - Contribution to designing it
    - Selecting patients at risk- criteria for including or excluding people from the list and whether this changed
    - Providing feedback
    - Putting patients in touch with support (eg food parcels)

4. What do you think of the shielding programme?
    - Has it been implemented as planned?
    - Positive aspects – for patients, for health service
    - Negative aspects – for patients, for health service
    - Have things changed over time?

    How did the way you delivered healthcare change for patients who were shielding?

5. We have already talked to patients who were part of the shielding programme. Here are two of their stories. *Share written vignettes, with names/details changed*.
    - Do these resonate with you? Is there anything in them which surprises you?
    - Have you discussed the shielding programme with any of your patients? What did they tell you?

For peer review only - http://bmjopen.bmj.com/site/about/guidelines.xhtml

6. Are you aware of any health related risks that your patients have experienced associated with the shielding programme?

7. Do you have any suggestions for any way it could be improved?

For peer review only

For peer review only - http://bmjopen.bmj.com/site/about/guidelines.xhtml

TREND Statement Checklist

Paper Section/ Topic	Item No	Descriptor	Reported? ✓	Pg #
**Title and Abstract**				
Title and Abstract	1	• Information on how unit were allocated to interventions	√	4
		• Structured abstract recommended	√	4
		• Information on target population or study sample	√	4
**Introduction**				
Background	2	• Scientific background and explanation of rationale	√	6-7
		• Theories used in designing behavioral interventions	√	6-7
**Methods**				
Participants	3	• Eligibility criteria for participants, including criteria at different levels in recruitment/sampling plan (e.g., cities, clinics, subjects)	√	8-13
		• Method of recruitment (e.g., referral, self-selection), including the sampling method if a systematic sampling plan was implemented	√	8-13
		• Recruitment setting	√	8-13
		• Settings and locations where the data were collected	√	8-13
Interventions	4	• Details of the interventions intended for each study condition and how and when they were actually administered, specifically including:	√	9
		○ Content: what was given?	√	9
		○ Delivery method: how was the content given?	√	9
		○ Unit of delivery: how were the subjects grouped during delivery?	√	9
		○ Deliverer: who delivered the intervention?	√	9
		○ Setting: where was the intervention delivered?	√	9
		○ Exposure quantity and duration: how many sessions or episodes or events were intended to be delivered? How long were they intended to last?	√	9
		○ Time span: how long was it intended to take to deliver the intervention to each unit?	√	9
		○ Activities to increase compliance or adherence (e.g., incentives)	√	6 + 9
Objectives	5	• Specific objectives and hypotheses	√	8
Outcomes	6	• Clearly defined primary and secondary outcome measures	√	10-13
		• Methods used to collect data and any methods used to enhance the quality of measurements	√	8-13
		• Information on validated instruments such as psychometric and biometric properties	√	10
Sample Size	7	• How sample size was determined and, when applicable, explanation of any interim analyses and stopping rules	√	10
Assignment Method	8	• Unit of assignment (the unit being assigned to study condition, e.g., individual, group, community)	√	10
		• Method used to assign units to study conditions, including details of any restriction (e.g., blocking, stratification, minimization)	√	10
		• Inclusion of aspects employed to help minimize potential bias induced due to non-randomization (e.g., matching)	√	10

For peer review only

Blinding (masking)	9	• Whether or not participants, those administering the interventions, and those assessing the outcomes were blinded to study condition assignment; if so, statement regarding how the blinding was accomplished and how it was assessed.	n/a	n/a
Unit of Analysis	10	• Description of the smallest unit that is being analyzed to assess intervention effects (e.g., individual, group, or community)	√	10
		• If the unit of analysis differs from the unit of assignment, the analytical method used to account for this (e.g., adjusting the standard error estimates by the design effect or using multilevel analysis)	n/a	n/a
Statistical Methods	11	• Statistical methods used to compare study groups for primary methods outcome(s), including complex methods of correlated data	√	13-14
		• Statistical methods used for additional analyses, such as a subgroup analyses and adjusted analysis	√	13-15
		• Methods for imputing missing data, if used	√	13
		• Statistical software or programs used	√	13-14
Results √				
Participant flow	12	• Flow of participants through each stage of the study: enrollment, assignment, allocation, and intervention exposure, follow-up, analysis (a diagram is strongly recommended)	√	25
		○ Enrollment: the numbers of participants screened for eligibility, found to be eligible or not eligible, declined to be enrolled, and enrolled in the study	n/a	n/a
		○ Assignment: the numbers of participants assigned to a study condition	√	10
		○ Allocation and intervention exposure: the number of participants assigned to each study condition and the number of participants who received each intervention	√	10
		○ Follow-up: the number of participants who completed the follow-up or did not complete the follow-up (i.e., lost to follow-up), by study condition	√	13
		○ Analysis: the number of participants included in or excluded from the main analysis, by study condition	√	13
		• Description of protocol deviations from study as planned, along with reasons	n/a	n/a
Recruitment	13	• Dates defining the periods of recruitment and follow-up	√	10
Baseline Data	14	• Baseline demographic and clinical characteristics of participants in each study condition	√	10-14
		• Baseline characteristics for each study condition relevant to specific disease prevention research	√	10-14
		• Baseline comparisons of those lost to follow-up and those retained, overall and by study condition	√	10-14
		• Comparison between study population at baseline and target population of interest	√	10-14
Baseline equivalence	15	• Data on study group equivalence at baseline and statistical methods used to control for baseline differences	√	10
Numbers analyzed	16	• Number of participants (denominator) included in each analysis for each study condition, particularly when the denominators change for different outcomes; statement of the results in absolute numbers when feasible	√	10
		• Indication of whether the analysis strategy was “intention to treat” or, if not, description of how non-compliers were treated in the analyses	√	13

Outcomes and estimation	17	• For each primary and secondary outcome, a summary of results for each estimation study condition, and the estimated effect size and a confidence interval to indicate the precision	√	9-15
		• Inclusion of null and negative findings	√	9-15
		• Inclusion of results from testing pre-specified causal pathways through which the intervention was intended to operate, if any	√	9-15
Ancillary analyses	18	• Summary of other analyses performed, including subgroup or restricted analyses, indicating which are pre-specified or exploratory	√	9-15
Adverse events	19	• Summary of all important adverse events or unintended effects in each study condition (including summary measures, effect size estimates, and confidence intervals)	√	9-15
DISCUSSION				
Interpretation	20	• Interpretation of the results, taking into account study hypotheses, sources of potential bias, imprecision of measures, multiplicative analyses, and other limitations or weaknesses of the study	√	9-15
		• Discussion of results taking into account the mechanism by which the intervention was intended to work (causal pathways) or alternative mechanisms or explanations	√	9-15
		• Discussion of the success of and barriers to implementing the intervention, fidelity of implementation	√	9-15
		• Discussion of research, programmatic, or policy implications	√	7-8, 16
Generalizability	21	• Generalizability (external validity) of the trial findings, taking into account the study population, the characteristics of the intervention, length of follow-up, incentives, compliance rates, specific sites/settings involved in the study, and other contextual issues	√	7-8
Overall Evidence	22	• General interpretation of the results in the context of current evidence and current theory	√	16

From: Des Jarlais, D. C., Lyles, C., Crepaz, N., & the Trend Group (2004). Improving the reporting quality of nonrandomized evaluations of behavioral and public health interventions: The TREND statement. *American Journal of Public Health*, 94, 361-366. For more information, visit: http://www.cdc.gov/trendstatement/

For peer review only
